# Inflammogenic effect of polyacrylic acid in rat lung following intratracheal instillation

**DOI:** 10.1186/s12989-022-00448-z

**Published:** 2022-01-21

**Authors:** Chinatsu Nishida, Taisuke Tomonaga, Hiroto Izumi, Ke-Yong Wang, Hidenori Higashi, Toru Ishidao, Jun-ichi Takeshita, Ryohei Ono, Kazuki Sumiya, Shota Fujii, Shinichi Mochizuki, Kazuo Sakurai, Kei Yamasaki, Kazuhiro Yatera, Yasuo Morimoto

**Affiliations:** 1 Department of Respiratory Medicine, University of Occupational and Environmental Health, Japan. 1-1 Iseigaoka, Yahata-nishi-ku, Kitakyushu, Fukuoka 807-8555 Japan; 2 Department of Occupational Pneumology, Institute of Industrial Ecological Sciences, University of Occupational and Environmental Health, Japan. 1-1 Iseigaoka, Yahata-nishi-ku, Kitakyushu, Fukuoka 807-8555 Japan; 3 Shared-Use Research Center, School of Medicine, University of Occupational and Environmental Health, Japan, 1-1 Iseigaoka, Yahata-nishi-ku, Kitakyushu, Fukuoka 807-8555 Japan; 4 Department of Environmental Health Engineering, Institute of Industrial Ecological Sciences, University of Occupational and Environmental Health, Japan, 1-1 Iseigaoka, Yahata-nishi-ku, Kitakyushu, Fukuoka 807-8555 Japan; 5 Department of Environmental Management, School of Health Sciences, University of Occupational and Environmental Health, Japan, 1-1 Iseigaoka, Yahata-nishi-ku, Kitakyushu, Fukuoka 807-8555 Japan; 6 Research Institute of Science for Safety and Sustainability, National Institute of Advanced Industrial Science and Technology (AIST), Tsukuba, Japan. 16-1 Onogawa, Tsukuba, Ibaraki 305-8569 Japan; 7 Department of Chemistry and Biochemistry, The University of Kitakyushu, 1-1, Hibikino, Wakamatsu-ku, Kitakyushu, Fukuoka 808-0135 Japan

**Keywords:** Cross-linked polyacrylic acid (CL-PAA), Organic chemicals, Pulmonary toxicity

## Abstract

**Background:**

Some organic chemicals are known to cause allergic disorders such as bronchial asthma and hypersensitivity pneumonitis, and it has been considered that they do not cause irreversible pulmonary fibrosis. It has recently been reported, however, that cross-linked acrylic acid-based polymer, an organic chemical, might cause serious interstitial lung diseases, including pulmonary fibrosis. We investigated whether or not intratracheal instillation exposure to cross-linked polyacrylic acid (CL-PAA) can cause lung disorder in rats.

**Methods:**

Male F344 rats were intratracheally instilled with dispersed CL-PAA at low (0.2 mg/rat) and high (1.0 mg/rat) doses, and were sacrificed at 3 days, 1 week, 1 month, 3 months and 6 months after exposure to examine inflammatory and fibrotic responses and related gene expressions in the lungs. Rat lungs exposed to crystalline silica, asbestos (chrysotile), and NiO and CeO_2_ nanoparticles were used as comparators.

**Results:**

Persistent increases in total cell count, neutrophil count and neutrophil percentage, and in the concentration of the cytokine-induced neutrophil chemoattractant (CINC)-1, CINC-2 and C-X-C motif chemokine 5 (CXCL5), which correlated with lung tissue gene expression, were observed in bronchoalveolar lavage fluid (BALF) from 3 days until at least 1 month following CL-PAA intratracheal instillation. Persistent increases in heme oxygenase-1 (HO-1) in the lung tissue were also observed from 3 days to 6 months after exposure. Histopathological findings of the lungs demonstrated that extensive inflammation at 3 days was greater than that in exposure to silica, NiO nanoparticles and CeO_2_ nanoparticles, and equal to or greater than that in asbestos (chrysotile) exposure, and the inflammation continued until 1 month. Fibrotic changes also progressed after 1 month postexposure.

**Conclusion:**

Our results suggested that CL-PAA potentially causes strong neutrophil inflammation in the rat and human lung.

**Supplementary Information:**

The online version contains supplementary material available at 10.1186/s12989-022-00448-z.

## Background

Inorganic chemicals such as asbestos and crystalline silica are known to cause irreversible interstitial pulmonary fibrotic lesions such as pneumoconiosis. Organic chemicals, conversely, cause allergic lung diseases such as bronchial asthma and hypersensitivity pneumonitis, but it is believed that they do not cause irreversible interstitial lesions like pulmonary fibrosis. Recent reports from South Korea, however, have shown that the organic chemical polyhexamethylene guanidine phosphate (PHMG-p), used as a humidifier disinfectant, caused lung disorders, including interstitial lung disease and acute respiratory distress syndrome (ARDS), in 5955 people [1, 2]. Occupational and environmental exposure to organic chemicals (e.g., exposure to wood dust, livestock and vegetable dust/animal dust) has also been identified as a potential risk factor for developing irreversible pulmonary fibrosis with very poor prognosis (5-year survival rate of 20 to 30%) [3]. It is fully conceivable, therefore, that exposure to other organic chemicals could also lead to irreversible pulmonary fibrosis. It has been reported that some workers at small and medium-sized enterprises in Japan that handle cross-linked acrylic acid-based polymers (CL-PAA) suffered from progressive lung disorder (Morimoto Y, et al. submitted), and the working group on occupational accident diseases of the Ministry of Health, Labour and Welfare compiled a report. According to governmental reports [4], in a workplace where CL-PAA were handled, 6 out of tens of workers who had used a powder of the polymers developed pneumoconiosis, emphysema or pneumothorax. All of those workers were in their 20–40 s, and many of them developed lung disease in about two years after the beginning of exposure. According to the dust concentration by the researchers who investigated there, the maximum personal exposure concentration of respirable dust was 2.1 mg/m^3^ (7.8 mg/m^3^ as inhalable dust) at 8 h-Time Weighted Average (8 h-TWA), and the personal exposure concentration was high in the extreme, especially in the work of adding CL-PAA-based to the hopper (41.8 mg/m^3^ as inhalable dust).

CL-PAA have been used as intermediates in the manufacture of pharmaceuticals and cosmetics. Although pneumoconiosis caused by inorganic dusts has been known to progress slowly over 20 years or more after exposure to the causative substance [5–7], this organic chemical induced lung disorders in a surprisingly short period after exposure, and the disorders progressed significantly faster than pneumoconiosis due to inorganic chemicals such as asbestos and crystalline silica. Based on the above, the Japanese working group speculates that there is a causal relationship between exposure to CL-PAA and lung disorder, but so far there has not been sufficient evidence of lung disorder caused by CL-PAA. The products made by CL-PAA are considered to develop no pulmonary toxicities because of no powder forms.

The putative mechanism of lung disorder caused by inorganic chemicals is that the inhaled chemicals deposit in the lung and cause persistent inflammation, and eventually lead to the formation of chronic and irreversible lesions such as pulmonary fibrosis and tumors [8–12]. Asbestos and crystalline silica, which have high pulmonary toxicity, have been reported to cause persistent inflammation in the lungs, resulting in pulmonary fibrosis and tumorigenesis [13, 14]. Thus, in lung disorder caused by inhalable dust, persistent lung inflammation is considered to be an important process in the induction and progression of chronic and irreversible lesions in the lung [8, 10–12].

In order to investigate lung disorder caused by CL-PAA, we performed intratracheal instillation of CL-PAA in a rat model and analyzed the inflammatory and fibrotic responses in the lung. Polyacrylic acid (PAA) is a basic structure among CL-PAA, and is an acrylic acid-based polymer obtained by homopolymerizing acrylic acid monomer. CL-PAA is widely used in various fields not only in Japan but also all over the world, and its production and quantity of import have been increasing over the years in Japan [15].

## Results

### Characterization of CL-PAA

The fundamental characteristics of CL-PAA are summarized in Tables 1 and 2. Briefly, the polymer used in our study had a weight average molecular weight (M_W_) of 6.49 million. The secondary particle diameter (the median or mode diameter) and hydrodynamic diameter were 3.00 µm (the median diameter) and 2.31 μm (the mode diameter), and 1.62 µm, respectively. The mass concentration in dispersion condition was adjusted to 2.5 mg/mL and the expected number concentration in dispersion condition was 4.28 × 10^9^ particles/mL. The effective density in dispersion with or without solvent were 1.08 g/mL and 0.26 g/mL, respectively. The mass median aerodynamic diameter (MMAD) was 0.92 µm. The mass concentration in aerosol condition was adjusted to 1.76 mg/m^3^ and the number concentration in aerosol condition was 3.01 × 10^6^ particles/m^3^. The effective density of aerosol condition was 1.43 g/mL.

**Table 1:** Physiochemical characterization of the polymer used in the present study

Name	Polyacrylic acid	Structural formula
CAS number		9003-01-04
Bulk	Purity	≦ 100% (Benzene 0.5%)
	Molecular weight	
	Weight average molecular weight (M_W_)	6,490,000
	Viscosity average molecular weight (Mv)	3,000,000 (average)
	Cross-liking	~ 0.1%
	Appearance	Solid, white powdered
	Odor	None
Suspension	*Secondary particle diameter	3.00 µm (median), 2.31 µm (mode)
	Hydrodynamic diameter	1.62 µm (average)
	**Effective density	1.08 (with solvent)
	***Effective density	0.26 (without solvent)
Aerosol	Mass median aerodynamic diameter	0.92 µm
	****Effective density	1.43 g/mL

*Secondary particle diameter: the particle diameter of agglomerate. Mode diameter: the most frequent particle size

**The effective density (g/mL) (with solvent) ρ_Eff_in dispersion with solv._$=\frac{({\rho }_{poly} {V}_{poly}) +({\rho }_{solv} {V}_{solv})}{V_{total}}$

$$=\frac{\left(1.43 \times \frac{\pi }{6} {0.92}^{3}\right) + \left(1.00 \times \frac{\pi }{6} \left({1.62}^{3 }- {0.92}^{3}\right)\right)}{\frac{\pi }{6} {1.62}^{3}}$$

$$=$$ 1.08 g/mL

here, ρ_Eff in dispersion with solv._: effective density in dispersion with solvent, ρ_poly_: density of polymer (= effective density of polymer), ρ_solv_: density of solvent (= density of water), *V*_poly_: volume of polymer, *V*_solv_: volume of solvent), *V*_total_: volume of swelled polymer (= *V*_poly_ + *V*_solv_).

***The effective density (g/mL) (without solvent)

ρ_Eff_in dispersion without solv._ $=$ $\frac{Mass\, concentration \,({\rm mg}/{\rm mL})}{Volume \,concentration \,({\rm mL}/{\rm mL})}$

$$=$$ $$\frac{2.5}{9.52 \times {10}^{-3}}$$

$$=$$ 0.26 g/mL

here, ρ_Eff in dispersion without solv._: effective density in dispersion without solvent

****The effective density (g/mL) (aerosol)

ρ_Eff_aerosol_ $$=$$ $$\frac{Mass \,concentration \,({\rm g}/{\rm m}^{3})}{Volume \,concentration \,({\rm mL}/{\rm mL})}$$

$$=\frac{1.76 \times {10}^{-3} }{9. 52}$$

$$=$$ 1.43 g/mL

here, ρ_Eff aerosol._: effective density of aerosol

**Table 2:** Aerodynamic, hydrodynamic diameters and effective densities of CL-PAA

	Mass concentration	Diameter [µm]	Number concentration	Volume concentration	Effective density [g/mL]
Aerosol condition	1.76 mg/m^3^-air	0.92 (aerodynamic)	3.01 × 10^6^ particles/L-air	1.23 × 10^–3^ mL/m^3^-air	1.43
Dispersion condition	2.5 mg/mL-solv	1.62 (hydrodynamic)	4.28 × 10^9^ particles/mL-solv	9.52 × 10^–3^ mL/mL-solv	0.26 (without solvent)
Dispersion condition	2.5 mg/mL-solv	1.62 (hydrodynamic)	–	–	1.08 (with solvent)

**Fig. 1:**
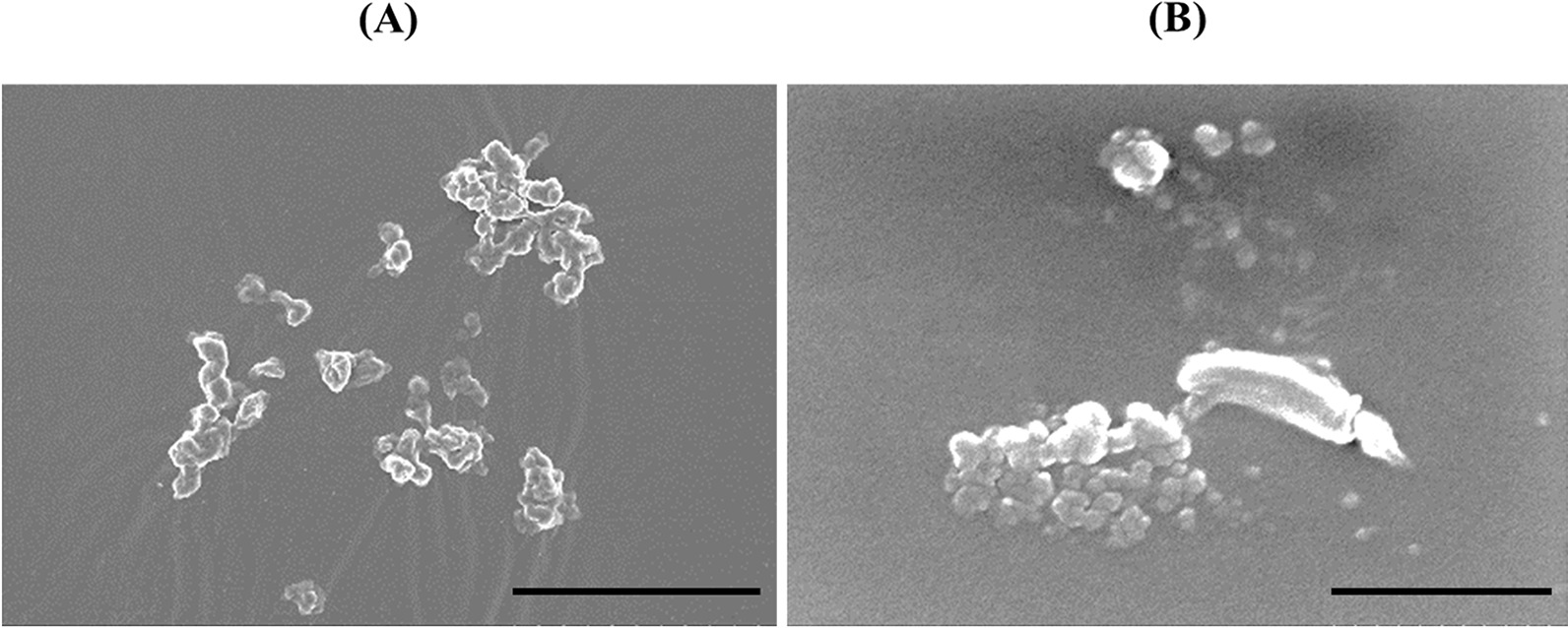
SEM images of the CL-PAA. Both the bulk polymer powder (**A**) and the suspended polymer in distilled water (**B**) made up agglomerates (internal scale bar = 20 μm for (**A**) and 0.5 µm for (**B**))

Both the bulk polymer (Fig. 1A) and the dispersed polymer in the solution (Fig. 1B) formed agglomerates.

Endotoxin was not detected in the suspension of CL-PAA (the data is shown in Additional file 3).

### Body and lung weights

**Fig. 2:**
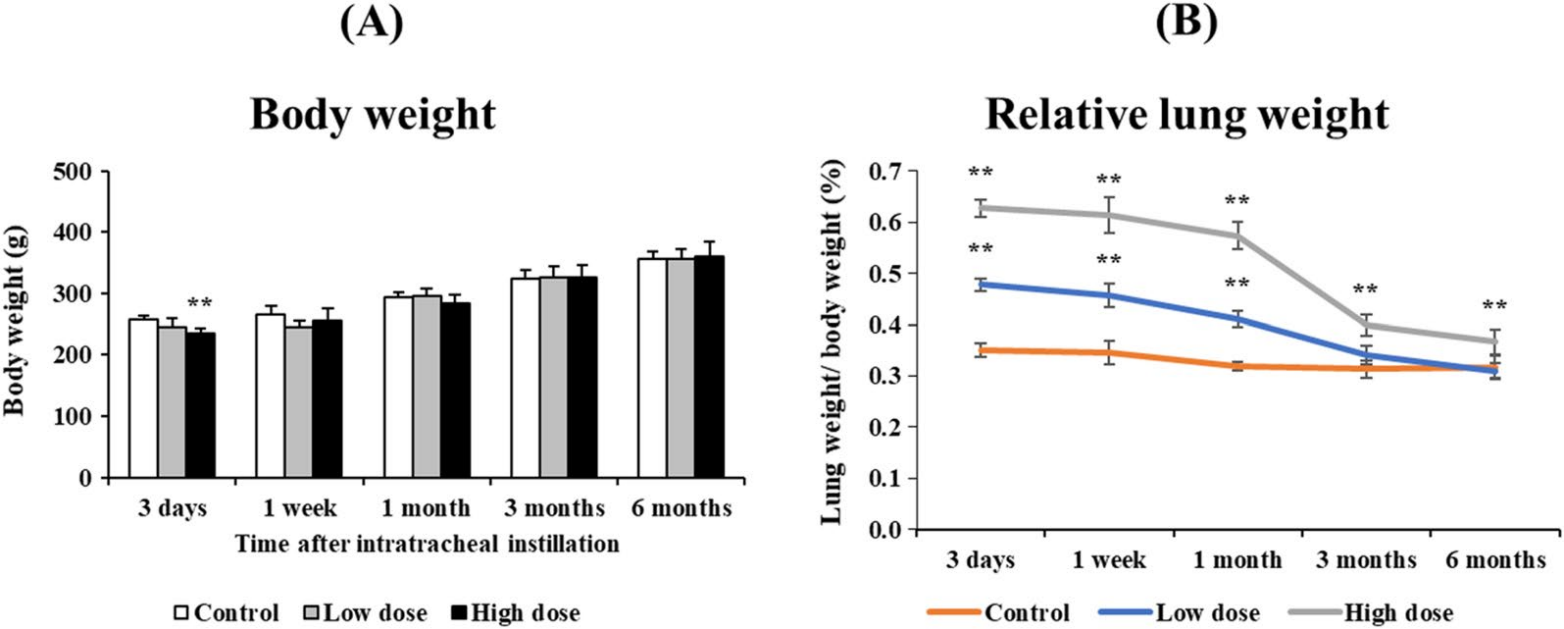
Body weight and relative lung after the instillation. **A** Time course of changes in the body weights of rats in each group. **B** Relative weight of the whole lung was calculated as a ratio of whole lung weight (g) to body weight (g) for each rat. The relative lung weight in the exposed groups were generally significantly heavier than in the control group in a dose dependent-manner throughout the observation period. Data are presented as mean ± SE (*p < 0.05, **p < 0.01)

**Fig. 3:**
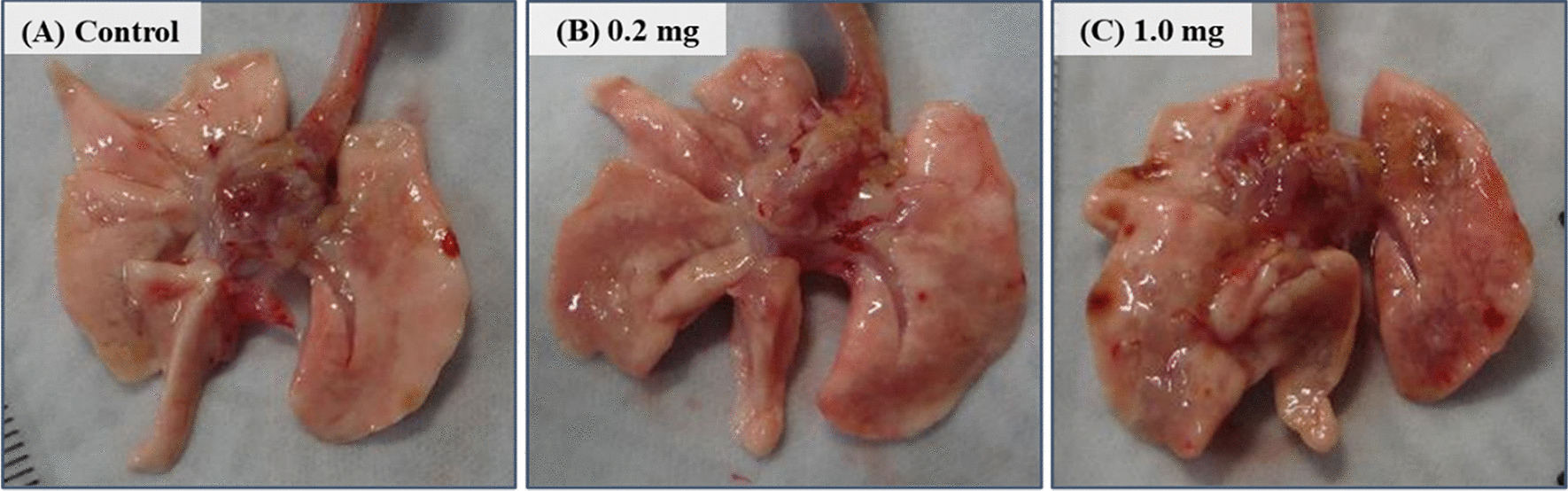
Gross findings at 3 days after the instillation. The lungs in the exposed groups showed ballooning at 3 days following intratracheal instillation. **A** control group. **B** 0.2 mg-exposure group. **C** 1.0 mg-exposure group

There were no significant changes in body weight in all of the groups, except for in the 1.0 mg-exposure group at 3 days after the instillation (Fig. 2A). The relative lung weight (lung weight/body weight) increased in a dose dependent-manner during the observation period (Fig. 2B). The lungs were edematous and mottled, especially in the 1.0 mg-exposure group, at 3 days after the instillation (Fig. 3).

### Cell analysis and lactate dehydrogenase (LDH) activity in bronchoalveolar lavage fluid (BALF)

**Fig. 4:**
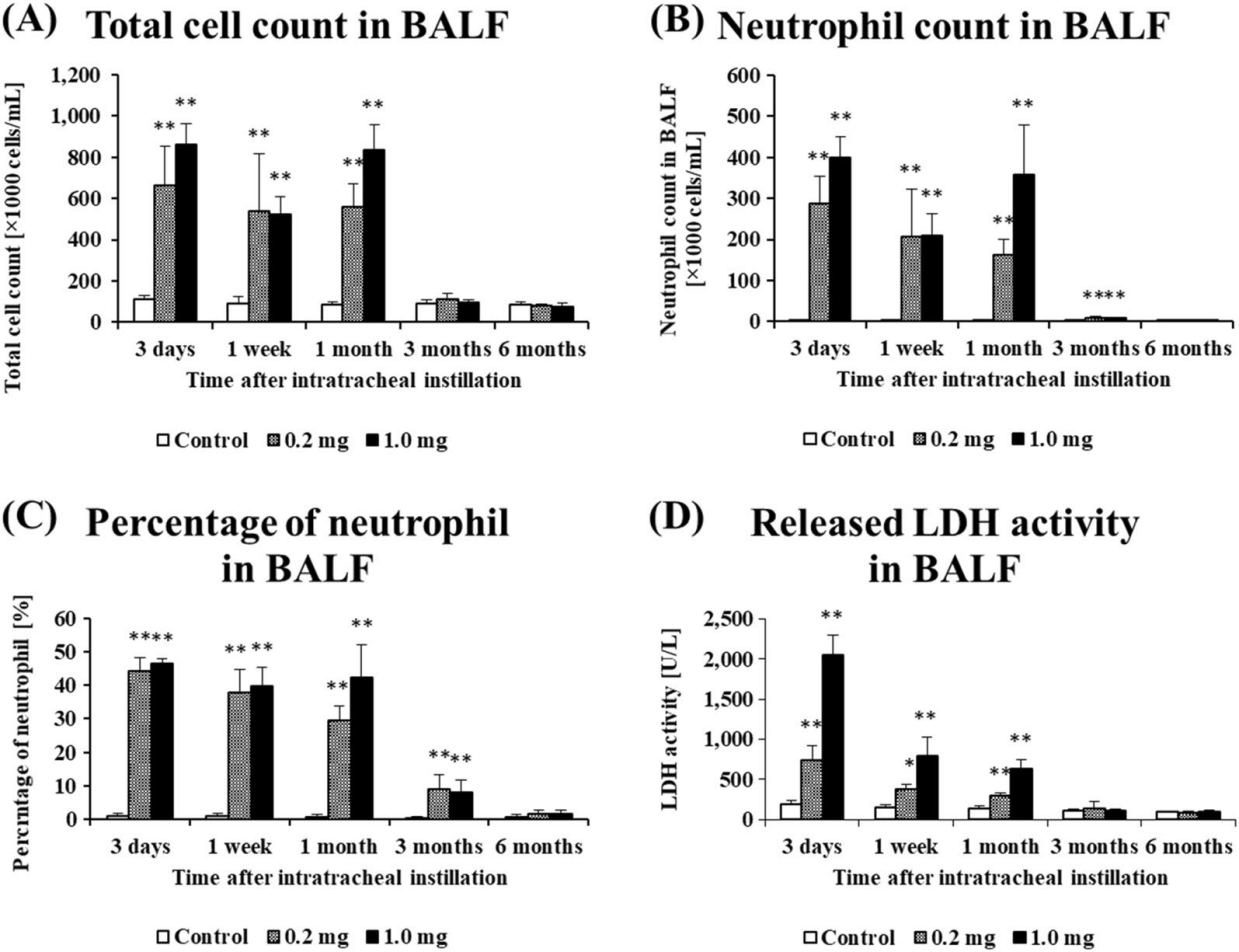
Analysis of cell number and released LDH activity in BALF following intratracheal instillation. **A** total cell number in BALF. **B** neutrophil count in BALF. **C** percentage of neutrophil in BALF. **D** released LDH activity in BALF. Inflammatory cell counts and LDH activity in BALF in the exposed groups were higher than those in the control group in a dose dependent-manner during 1 or 3 months after the instillation. Data are presented as mean ± SE (*p < 0.05, **p < 0.01)

**Fig. 5:**
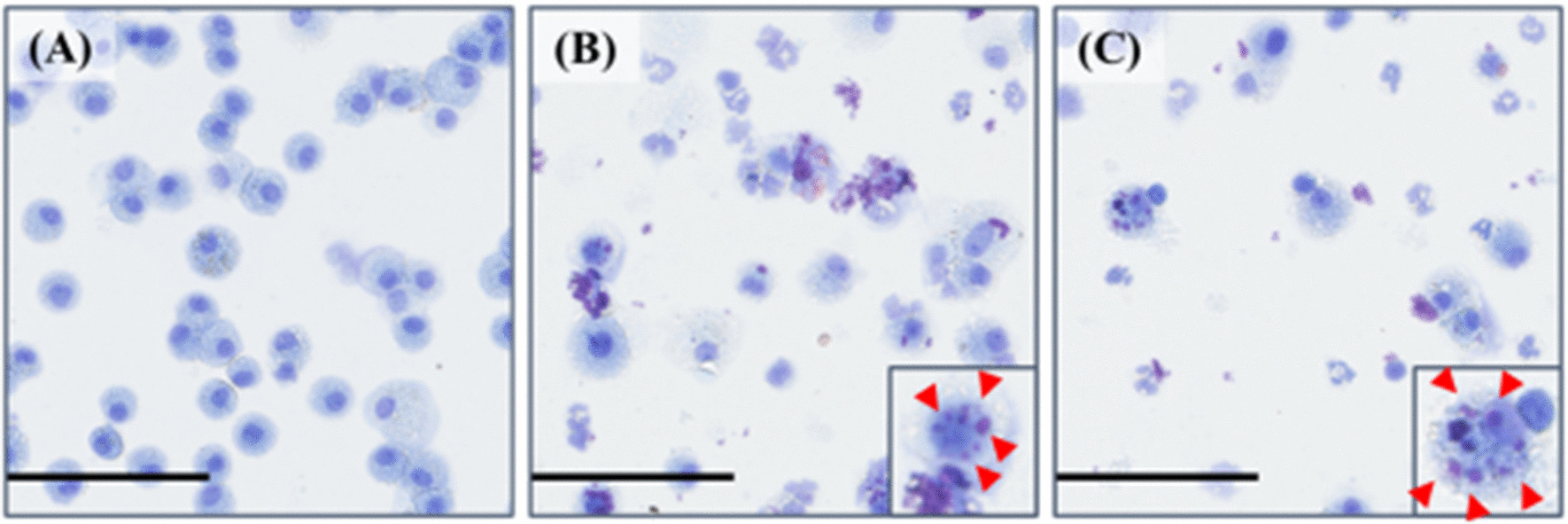
Cells in BALF at 3 days after the instillation. The images of polymer phagocytosis by macrophages (arrow heads) are shown in the insets. **A** control group. **B** 0.2 mg-exposure group. **C** 1.0 mg-exposure group (internal scale bar = 100 μm for all)

Figure 4 shows the results of inflammatory cell counts and LDH activity, an index of cell injury, in BALF. There was a statistically significant increase in the number of total cells in the 0.2 mg and 1.0 mg-exposure groups from 3 days to 1 month after exposure compared to the control group (Fig. 4A). There were significant increases in the number of neutrophils (Fig. 4B) and the percentage of neutrophils (Fig. 4C) from 3 days to 3 months after exposure. Optical microscopic images of the BALF findings at 3 days after the instillation demonstrated that there were many neutrophils and many macrophages that had phagocytized CL-PAA in the exposure groups (Fig. 5), and its findings persisted for 3 months after exposure. The results of released LDH activity in the 0.2 mg and 1.0 mg-exposure groups also showed statistically significant increases from 3 days to 1 month after exposure compared to the control group (Fig. 4D).

### Concentration of cytokine-induced neutrophil chemoattractant (CINC) and C-X-C motif chemokine (CXCL5) in BALF and concentration of heme oxygenase (HO)-1 in lung tissue

**Fig. 6:**
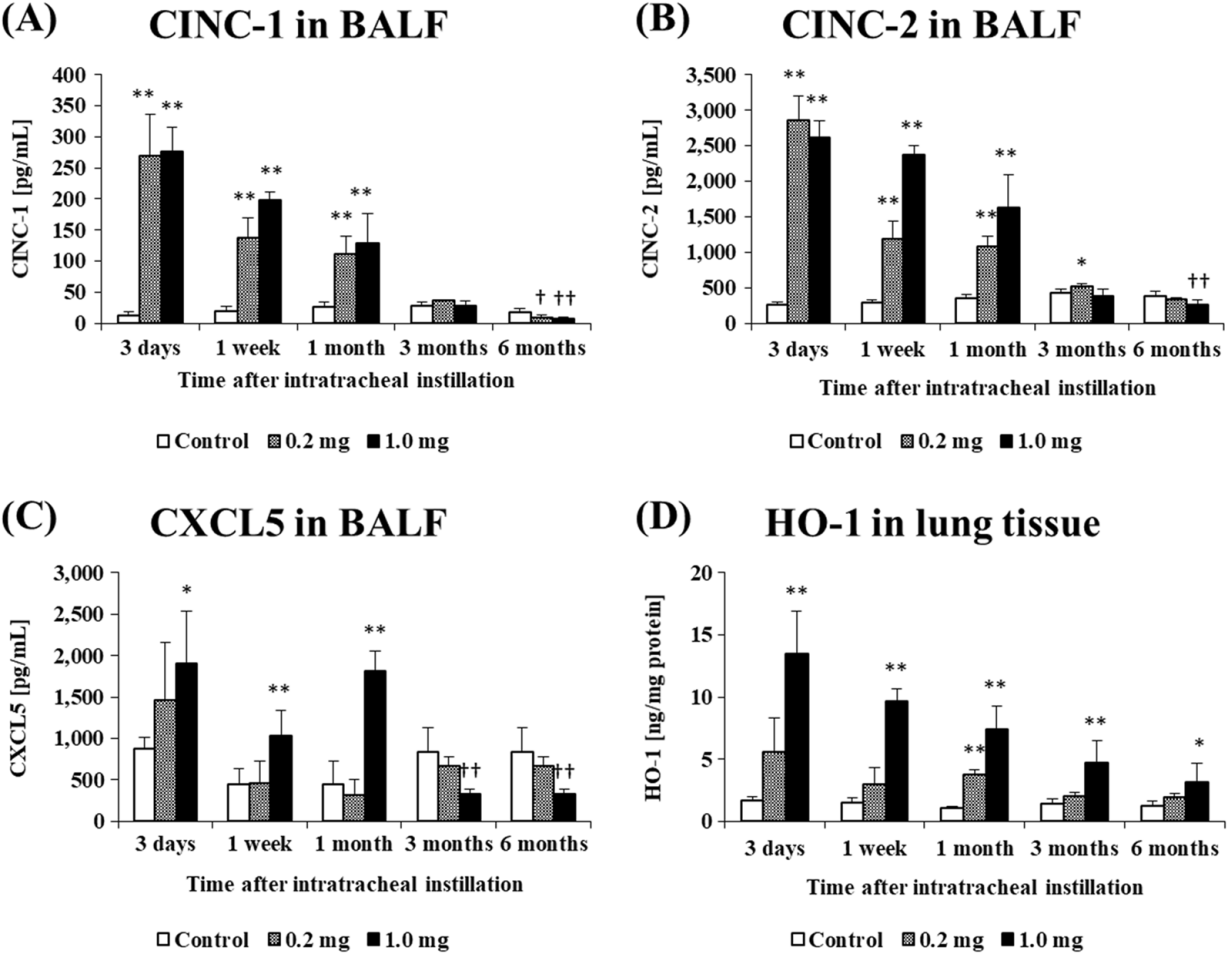
Analysis of cytokines in BALF and HO-1 in lung tissue following intratracheal instillation. **A** CINC-1/CXCL1 concentration in BALF. **B** CINC-2/CXCL3 concentration in BALF. **C** CXCL5 concentration in BALF. **D** HO-1 concentration in lung tissue. The expressions of CINC-1/CXCL1, CINC-2/CXCL3 and CXCL5 in BALF in the exposed groups were persistently higher compared to the control group in a dose dependent-manner during 1 month after the exposure. Data are presented as mean ± SE (*p < 0.05 and **p < 0.01 indicate that the values are significantly higher than control group. ^†^*p* < 0.05 and ^††^*p* < 0.01 indicate that the values are significantly lower than control group.)

Figure 6A–C shows the concentrations of CINC-1, CINC-2 and CXCL5 in BALF following the intratracheal instillation of CL-PAA. The concentrations of CINC-1, CINC-2 and CXCL5 increased persistently in a dose dependent-manner from 3 days until 1 month postexposure. The expression levels of these three chemokines in the exposed groups decreased with time, and no significant increase was observed after 3 months postexposure in general. Statistically significant persistent increases in the concentration of HO-1, which is an oxidative stress inducible protein and is suggested to be involved in pulmonary fibrosis, in the lung tissues exposed to CL-PPA were observed during the observation time.

### Gene expression analysis

Table 3 shows the number of genes, among 20,174 genes examined by cDNA microarray, sorted by the fold change of mRNA expression levels in lung tissue at 1 month in the 1.0 mg CL-PAA-exposure group compared to the control group. More than eightfold upregulated chemokine genes involved in “inflammatory response” are shown in Table 4. Among them, the most upregulated gene was *CXCL5* (also known as *C-X-C motif chemokine* 6 (*CXCL6*) in rats), and there was a 69.02-fold amount in the 1.0 mg CL-PAA-exposure group compared with the control group. Additional file 1: Table S1 shows the upregulated genes related to “inflammatory response” (Additional file 1: Table S1(A): 52 genes), “immune response” (Additional file 1: Table S1(B): 47 genes) and “response to oxidative stress” (Additional file 1: Table S1(C): 9 genes) with more than twofold upregulation compared to the control group. Moreover, genes related to “epithelial to mesenchymal transition (EMT)” and “positive regulation of EMT” are listed Additional file 1: Table S2 ((A): (1 gene) and (B): (3 genes)), respectively.

**Table 3:** Number of genes by mRNA expression level in the polymer-high dose group at one month

mRNA level (fold change of control)	Number of genes
*Up regulation*	
≧ twofold	788
2 ~ fourfold	620
4 ~ eightfold	110
≧ eightfold	58
*Down regulation*	
≦1/2-fold	668
1/2~1/4-fold	648
1/4~1/8-fold	17
≦1/8-fold	3

Table shows number of genes by mRNA expression level in the polymer-high dose group at one month after intratracheal instillation among 20,174 genes examined using cDNA microarray

**Table 4:** Description of chemokine genes related to ‘inflammatory response’ among 58 genes upregulated ≧ eightfold

Gene symbol	Gene description	Fold change
Inflammatory response		
GO: 0006954		
*CXCL5*	*Chemokine (C-X-C motif) ligand5*	69.02
*CXCL11*	*Chemokine (C-X-C motif) ligand11*	33.73
*CCL7*	*Chemokine (C-C motif) ligand7*	32.69
*CCL2*	*Chemokine (C-C motif) ligand2*	30.93
*CXCL13*	*Chemokine (C-X-C motif) ligand13*	29.57
*CCL1*	*Chemokine (C-C motif) ligand1*	20.32
*CCL9*	*Chemokine (C-C motif) ligand9*	16.24
*CXCL10*	*Chemokine (C-X-C motif) ligand10*	13.51
*CCL12*	*Chemokine (C-C motif) ligand12*	12.64

Upregulated chemokine genes among the genes involved in “inflammatory response” among 58 genes upregulated ≧ eightfold

The result of Kyoto Encyclopedia of Genes and Genomes (KEGG) pathway revealed that differentially expressed upregulated genes were particularly significant enrichment in 35 pathways (Additional file 1: Table S3), including “cytokine-cytokine receptor interaction” and “chemokine signaling pathway” (Table 5 and Additional file 2: Figure S2 (A) and (B)).

**Table 5:** Signaling pathways of differentially expressed upregulated genes at 1 month after exposure to 1.0 mg-CL-PAA (*p* < 0.05 and gene counts ≧ 2)

Category	Term	Count^1^	%^2^	*p*-value^3^	Benjamini^4^
KEGG_PATHWAY	Cytokine-cytokine receptor interaction	31	5	7.00E−12	1.30E−09
KEGG_PATHWAY	Chemokine signaling pathway	29	4.7	1.10E−11	1.30E−09

^1^“Count” means the number of genes involved in the term

^2^“%” means percentage of “involved genes”/“total genes”

^3,4^Fisher’s exact test and modified Fisher’s exact test is adopted to measure the gene-enrichment in annotation terms, respectively

The total number of genes analyzed was 618 genes in this analysis

### Micro-CT imaging

**Fig. 7.**
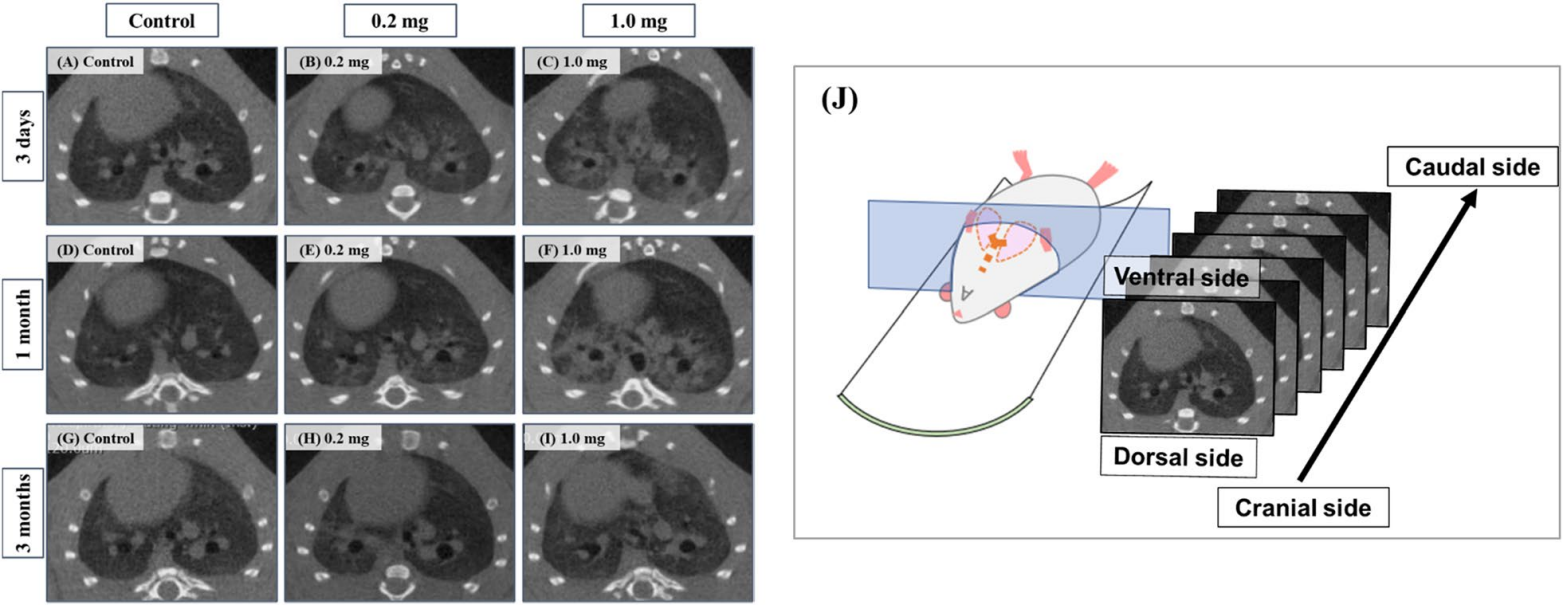
3D micro-CT imaging following intratracheal instillation and how to view the CT image and the relationship between the CT image and the anatomical position. At 3 days: **A** control group. **B** 0.2 mg-exposure group. **C** 1.0 mg-exposure group. At 1 month: **D** control group. **E** 0.2 mg-exposure group. **F** 1.0 mg-exposure group. At 3 monthss: **G** control group. **H** 0.2 mg-exposure group. **I** 1.0 mg-exposure group. Diffuse or centrilobular infiltration (red arrow) in both lungs was presented at 3 days and 1 month after the exposure in a dose-dependent manner. The finding was persisted during 1 month and improved at 3 months after the exposure. The relationship between the CT image and the anatomical position is shown at **J**

Micro-CT revealed diffuse or centrilobular infiltration in both lungs at 3 days and 1 month after the exposure in a dose-dependent manner (Fig. 7). An improvement of lung infiltrations was observed at 3 months as compared with those at 3 days and 1 month after the exposure.

### Histopathological features in the lung

**Fig. 8:**
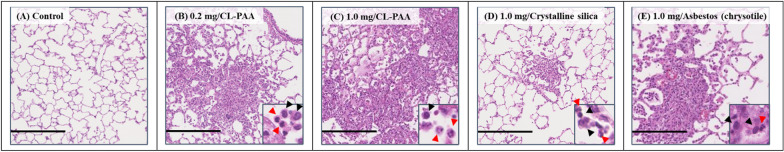
Histological findings at 3 days following the instillation. **A** distilled water as a negative control, **B** 0.2 mg CL-PAA-exposed lung, **C** 1.0 mg CL-PAA-exposed lung, **D** 1.0 mg crystalline silica-exposed lung, **E** 1.0 mg asbestos (chrysotile)-exposed lung, **F** 1.0 mg NiO nanoparticle-exposed lung, **G** 1.0 mg CeO_2_ nanoparticle-exposed lung. These are all HE stained specimens. There were inflammatory cells, mainly neutrophils (red arrow heads) and macrophages (black arrow heads), in the alveolar space in the exposed groups. Inflammatory cell infiltration was more pronounced in a dose dependent-manner, especially in the CL-PAA exposed groups, compared to in the other exposed groups (internal scale bar = 250 μm for all)

**Fig. 9:**
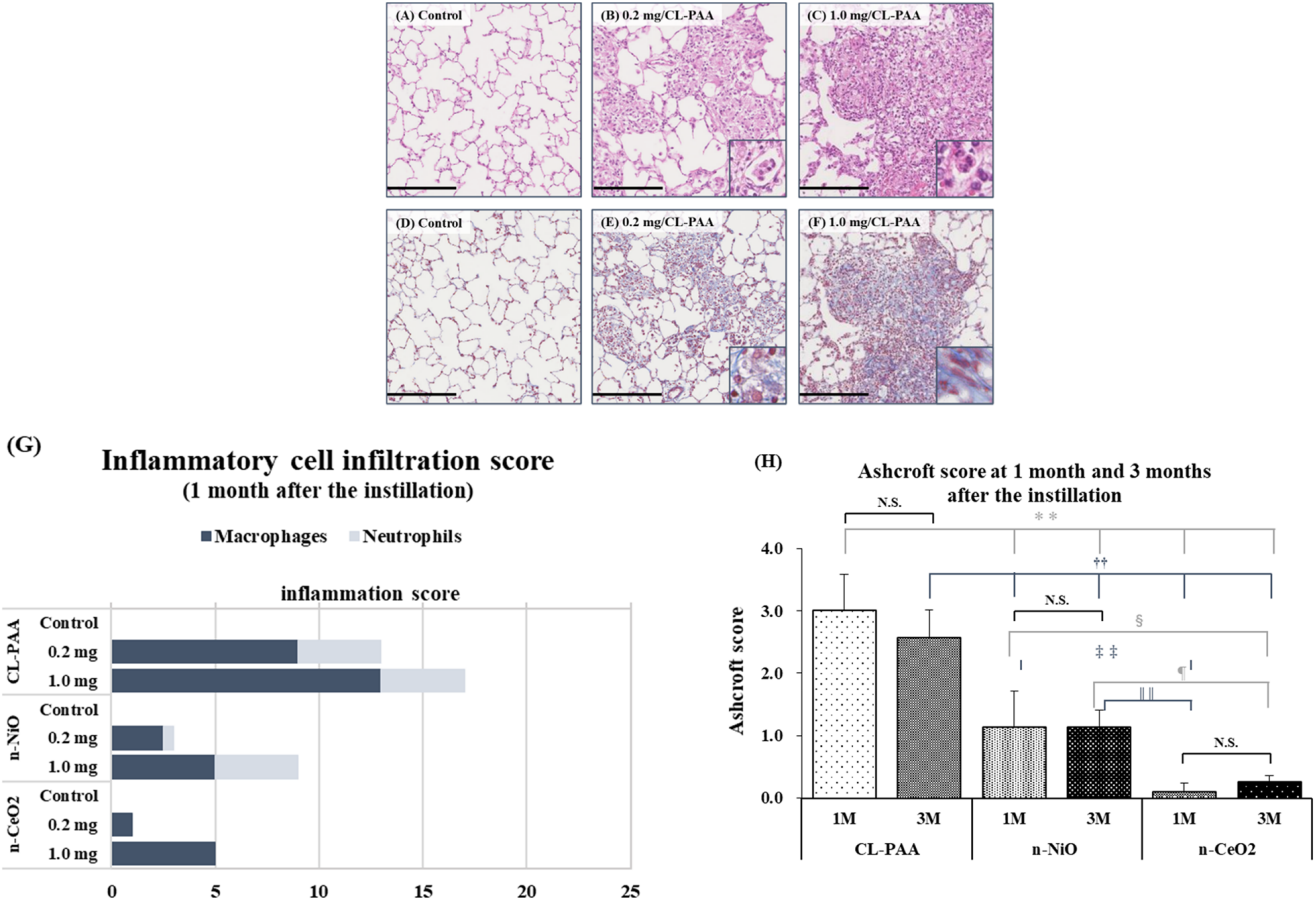
Histological findings at 1 month following the instillation and the evaluation of lung lesions. **A**–**C** and **D**–**F** show HE staining and MT staining images, respectively, at 1 month after the instillation of 0.2 mg or 1.0 mg-CL-PAA. Red and black arrow heads, and black arrows indicate neutrophils and macrophages, and collagen deposition, in the alveolar space or alveolar septa, respectively. Histological findings of NiO nanoparticles and CeO_2_ nanoparticles were shown in our previous report [16, 17]. **G** shows the inflammatory cell infiltration score in the 0.2 mg or 1.0 mg CL-PAA, NiO nanoparticles, or CeO_2_ nanoparticles-exposed lungs at 1 month after the instillation. The inflammatory cell infiltration was observed in dose dependent manner, and more prominent compared to the NiO nanoparticles or CeO_2_ nanoparticle. **H** shows the Ashcroft score in the 1.0 mg CL-PAA, NiO nanoparticles and CeO_2_ nanoparticles-exposed lungs at 1 month and 3 months after the instillation. Although pulmonary fibrosis induced was persisted in each exposed group during 1 month to 3 months after the instillation, it was more severe by CL-PAA than by NiO nanoparticles and CeO2 nanoparticles. The Ashcroft score of each control group was all zero. Data are presented as mean ± SE (**p < 0.01, ^††^*p* < 0.01, ^§^p < 0.05, ^‡‡^*p* < 0.01, ^¶^*p* < 0.05, ^||||^*p* < 0.01, *indicates that there is a significant difference in scores between the two groups. N.S. means no significance.) n-NiO and n-CeO_2_ indicate that NiO nanoparticles and CeO_2_ nanoparticles, respectively. One-way analysis of variance (ANOVA) followed by Turkey's test was used to appropriately to detect individual difference among the exposed groups

Representative histopathological findings in the lung at 3 days and 1 month after the instillation of CL-PAA are shown in Figs. 8A–C, H–J and 9, respectively. Inflammatory cell infiltrations into the alveoli, mainly neutrophils, were remarkable in the lung in a dose-dependent manner at 3 days after exposure to CL-PAA (Fig. 8A–C, H–J). The CL-PAA-induced inflammation in the lung at 3 days postexposure was greater than that in exposure to silica (Fig. 8D, K), NiO nanoparticles (Fig. 8F, M) and CeO_2_ nanoparticles (Fig. 8G, N), and equal to or greater than that in asbestos (chrysotile) exposure (Fig. 8E, L). The pathological features of lung inflammation persisted even 1 month after exposure, but there were no granulomas or formation of giant cells. While inflammatory cell infiltration was persistent, alveolar fibrosis was observed from 1 month (Fig. 9).

**Fig. 10:**
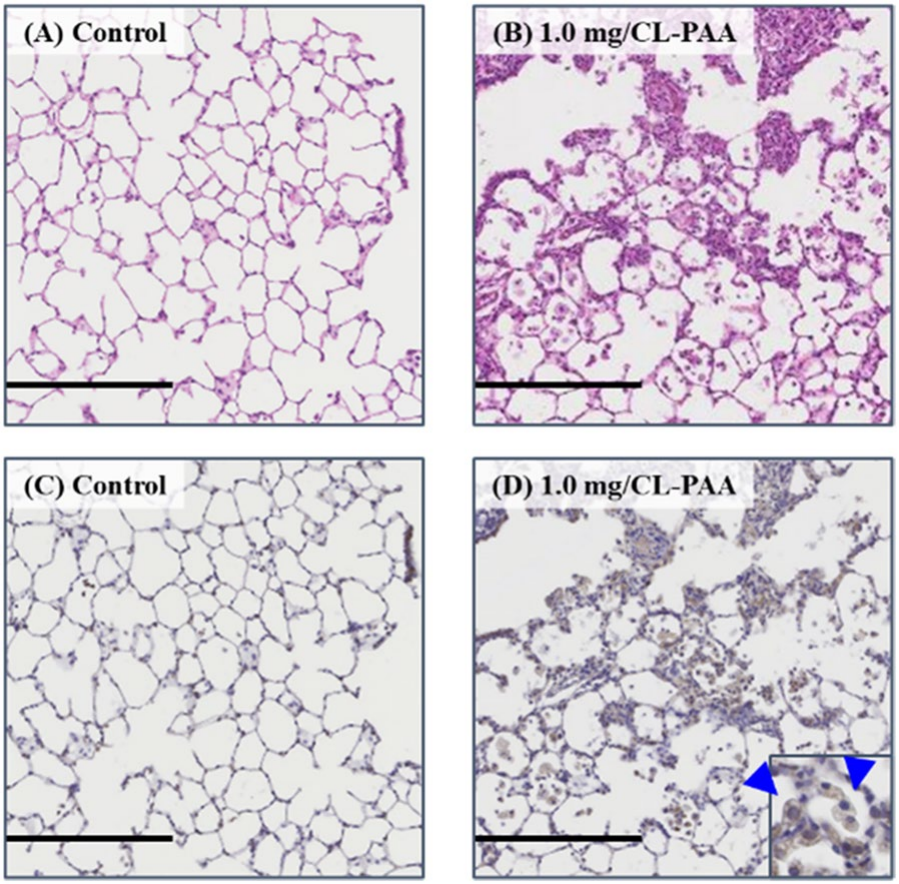
Representative images of CXCL5 immunostaining in lung tissue at 1 month after exposure to CL-PAA. **A** control lung (HE staining), **B** 1.0 mg CL-PAA-exposed lung (HE staining), **C** control lung (CXCL5 immunostaining), **D** 1.0 mg CL-PAA-exposed lung (CXCL5 immunostaining). Positive cells of CXCL5 immunostaining on 1.0 mg CL-PAA-exposed lung were mainly macrophages (blue arrow heads) (internal scale bar = 250 μm for all)

Lung immunohistochemistry at 1 month after exposure to 1.0 mg-CL-PAA demonstrated that the CXCL5-positive cells were macrophages around the neutrophil infiltration (Fig. 10).

**Fig. 11:**
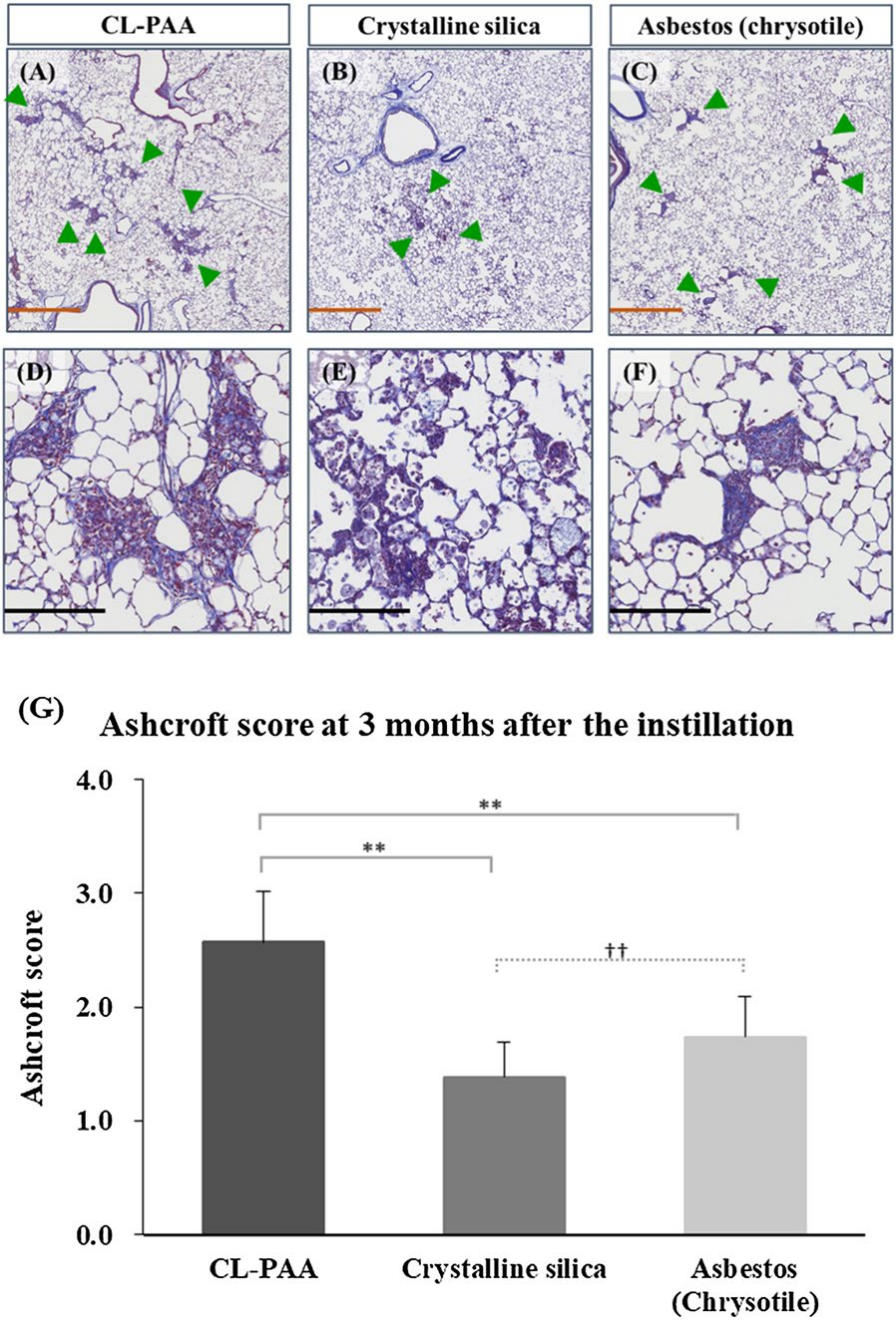
Histological findings and the Ashcroft score at 3 months following the instillation. **A**, **D** 1.0 mg CL-PAA-exposed lung, **B**, **E** 1.0 mg crystalline silica-exposed lung, **C**, **F** 1.0 mg asbestos (chrysotile)-exposed lung. **A**–**C** are low magnification images of **D**–**F**, respectively. Although pulmonary fibrosis (green arrow heads) was observed around the bronchiole region in all groups, it was more prominent in the CL-PAA group compared to the crystalline silica and asbestos (chrysotile) group (internal scale bar = 2.5 mm for brown, 250 μm for black) The Ashcroft score at 3 months after the instillation **G** showed that CL-PAA induced more severe pulmonary fibrosis than crystalline silica and asbestos (chrysotile). The Ashcroft score of each control group was all zero. Data are presented as mean ± SE (**p < 0.01 and ^††^*p* < 0.01,*indicates that there is a significant difference in the scores between the two groups, respectively.)

Pulmonary fibrosis was observed in the lungs 1 month after CL-PAA exposure. The degree of fibrosis was more severe compared to the lungs exposed to crystalline silica and asbestos (chrysotile) (Fig. 11).

## Discussion

The main findings obtained in the present study are as follows: (1) The CL-PAA caused severe lung inflammation and fibrosis. (2) The lung inflammation induced by CL-PAA occurred in a dose dependent-manner. (3) The HO-1 protein level in the lung tissue increased persistently during the observation period.

In the present study, there was marked neutrophil-based inflammatory cell infiltration in the alveoli in the lungs following intratracheal instillation of CL-PAA and it persisted until 1 month. We previously performed intratracheal instillations of various inorganic chemicals under the same experimental conditions of dose and observation period: crystalline silica, asbestos (chrysotile), and nanoparticles of nickel oxide (NiO) [16] and cerium oxide (CeO_2_) [17], which has high pulmonary toxicity among manufactured nanomaterials, and multi-walled carbon nanotube (MWCNT) [18] with lung tumorigenesis. Although all of these materials also induced persistent inflammation mainly due to neutrophil infiltration, the lung inflammation caused by CL-PAA was equal to or stronger than that of those materials. Actually, in the present study, we found more extensive inflammation in the lung than by the other substances with high pulmonary toxicity (Fig. 8), accompanied by a higher number and percentage of neutrophils in BALF [16, 17].

Reports on pulmonary toxicity induced by CL-PAA have been inconsistent, with some reports showing inflammation and fibrosis [19–22], and other reports not showing inflammation and fibrosis [21]. This difference in pulmonary toxicity may be related to the difference of the physicochemical properties of CL-PAA. There are some reports that physicochemical properties of respirable chemicals affect pulmonary toxicity. As for size of diameter of particles, intratracheal instillation of NiO nanoparticles induced severe and persistent inflammation of the lungs, whereas that of micron-sized particles did induce only transient mild inflammation [23, 24]. Furthermore, it is meaningful to clarify the physicochemical properties of CL-PAA and evaluate the pulmonary toxicity in the present study.

Regarding lung disorder caused by organic chemicals, there have been reports from South Korea of an animal model of exposure to PHMG-P, and severe lung inflammation which was mainly neutrophils was observed in all of the studies. Kim et al. performed an intratracheal instillation of PHMG-P (1.2 mg/kg BW, single exposure) in mice and observed them for 1 month after the exposure, and severe and persistent inflammation in the lung occurred until 1 month after the exposure [25]. Park et al. conducted an inhalation exposure to PHMG-P in rats (1.6 mg/m^3^, 6 h/day, 5 days/week, for 4 weeks), and severe lung inflammation was observed [26]. The clinical characteristics of PHMG-P induced lung disorder in humans are a short duration (within one year) of its use before the onset of lung disorder, rapid development of fibrosis following severe pneumonia, and a high mortality rate [27, 28]. In other reports, although the exposure doses were different from the present study, PHMG-p induced severe lung inflammation similarly to the present study, suggesting that CL-PAA has a high ability to cause lung inflammation.

The CINC-1 and CINC-2 concentrations in BALF increased persistently due to CL-PAA exposure. The CINC family are typical chemokines that induce and activate neutrophils and macrophages in rat lung. Intratracheal instillation of NiO and CeO_2_ nanoparticles under the same exposure dose as in the present study also showed an increase in CINC-1 and CINC-2 concentrations in BALF [16, 17, 29], and the level of increase due to CL-PAA exposure was almost the same (Additional file 2: Figure S1A, B). On the other hand, the gene expression of *CXCL5* was significantly higher, approximately 70-fold, as compared with the control group. CXCL5 is a CXC chemokine with a glutamate-leucine-arginine (ELR) motif (ELR + chemokine) that has strong chemotaxis and activation functions for lung neutrophils [30]. In our previous study, which was performed by the instillation of 1.0 mg-NiO and 1.0 mg-CeO_2_ nanoparticles, as in the present study, the gene expression of *CXCL5* in the lung tissue during the observation period of 3 days to 6 months was up to 20-fold higher than that in the control group [31]. The degree of neutrophil influx into the lungs by intratracheal instillation of CL-PAA was higher than that due to exposure to NiO and CeO_2_ nanoparticles. These results suggest that CXCL5 has an enhancing effect of neutrophil influx in lung disorder caused by CL-PAA, in addition to CINC-1 and CINC-2 being involved in neutrophil influx into the lung.

Intratracheal instillation of CL-PAA caused pulmonary fibrosis from 1 month after the exposure, and the extent was greater than that of crystalline silica and asbestos (chrysotile) in the lungs. This suggests that the CL-PAA used in the present study has a potent fibrotic ability. Similarly, as regards organic chemicals, exposure to PHMG-P induced more severe fibrosis than bleomycin at 1 month after the exposure by intratracheal instillation in a mice model [25]. Although the mechanism that caused the fibrosis due to exposure to CL-PAA is uncertain, in previous reports, it was shown that pulmonary fibrosis was partly dependent on angiogenesis, suggesting that CXCL5 was an important angiogenic factor in idiopathic pulmonary fibrosis [32]. Martinu T et al. [33] showed that pulmonary neutrophils and neutrophil chemoattractant including CXCL5 contributed to pulmonary fibrosis in a model of chronic pulmonary graft-versus-host disease, along with IL-17A. CXCL5 could not only induce lung inflammation, but also fibroblasts and myofibroblasts proliferation [34, 35]. The fibroblasts and myofibroblasts contribute to the pathogenesis of pulmonary fibrosis [36–41]. As one of the ideas about the origin of the fibroblasts and myoblasts in lung tissue, there is the concept that lung injury can induce epithelial cells to transition to a mesenchymal phenotype (EMT) [37, 41]. EMT is controlled by a network of signaling and transcriptional events mediated in part by TGF-β signaling [42, 43], and TGF-β increases α-SMA, one of mesenchymal markers [34]. Balli D et al.[34] revealed that lung inflammation and EMT through elevated expression of *CXCL5* and *CCL2* enhanced exacerbating with radiation-induced pneumonitis and pulmonary fibrosis. In our experiment, the upregulation of *CXCL5*, *CCL2* and EMT related genes such as *transforming growth factor, beta receptor 1 (Tgfbr1)* and collagen, type I, alpha 1 (*Colla1*) was also observed, suggesting that the similar mechanism worked. Altogether, we consider that increased expression of *CXCL5* may be involved in the development and persistence of pulmonary fibrosis by promoting EMT.

In the comprehensive gene analysis, the upregulation of HO-1 was higher among the group of “response to oxidative stress”, and 4.62 times compared to the control group. It was also found that there was a persistent increase in the concentration of HO-1 protein level in the lung tissue in the present study. HO-1 has been reported to be involved in the progression or the severity of pulmonary fibrosis [44, 45]. In our previous study, we observed a persistent increase in the HO-1 protein level in lung tissue exposed to NiO nanoparticles [24], similar to a persistent increase in CINC-1 and CINC-2 in BALF. In the analysis of the HO-1 protein level in BALF, a persistent increase was shown in intratracheal instillation of the same NiO nanoparticles [16]. On the other hand, the HO-1 in BALF increased transiently in an intratracheal instillation of titanium dioxide (TiO_2_) nanoparticles (Rutile) with low pulmonary toxicity [16]. The persistent increase in the HO-1 in the lung tissue in the present study is considered to reflect lung disorder. Furthermore, although we analyzed HO-1 in the lung tissue after the recovery of BALF, the level of HO-1 was higher than that in lung tissue exposed to NiO nanoparticles without recovery of BALF. CL-PAA exposure led to more extensive and severe lung inflammation and higher HO-1 levels in lung tissue than other particles with high pulmonary toxicity, suggesting that CL-PAA induced lung disorder through oxidative stress. It has been reported that oxidative stress made lung injury more progressive in knockout mice of class A scavenger receptors (SR-As) [46].

A limitation of this study is that, although intratracheal instillation studies can be useful for estimating the hazardous effects of inhalable chemicals, its exposure route is not physiological, in spite of the instillation of CL-PAA of a respirable size, unlike in inhalation studies. Therefore, inhalation studies are required to elucidate whether or not exposure to CL-PAA induces pulmonary inflammation and fibrosis.

## Conclusion

In the present study, we performed intratracheal instillation of CL-PAA in rats and examined lung inflammation and fibrosis in an observation period of 3 days to 6 months. There was remarkable and persistent lung inflammation from just after the exposure, leading to fibrosis 1 month later. Chemokines such as CXCL5, CINC-1, and CINC-2, and oxidative stress were considered to be involved in the lung inflammation induced by CL-PAA. Taken together, these results suggest that CL-PAA has a high potential of induction of lung disorder.

## Material and methods

### Sample polymer

CL-PAA (306223 Poly (acrylic acid)®): average Mv ~ 3,000,000) (Sigma-Aldrich Co. LLC., St. Louis, MO, USA) was used. The polymer was mixed with distilled water, slowly stirred for 40 min (Mag-Mixer MF820 or MD300, Yamato Scientific co., Ltd., Tokyo, Japan) and then ultrasonically dispersed at 23 kHz for 10 min (ASU-10D, Taiyo Canpany Co., Ltd., Osaka, Japan). The weight average molecular weight (M_W_) of the polymer was measured by multiangle light scattering coupled with field flow fractionation (FFF-MALS) (Wyatt Technology Europe GmbH, Dernbach, Rheinland-Pfalz, Germany) [47]. The secondary diameter in the testing suspension and the hydrodynamic diameter were measured by a total holographic counting system using xSight (Spheryx, Inc., New York, NY, USA) [48] and by the dynamic light scattering (DLS), based on the Stokes–Einstein relationship of Brownian motion [49] using DynaPro NanoStar (Wyatt Technology Corp., Santa Barbara, CA, USA), respectively. The effective density in dispersion without solvent was determined by dividing the mass concentration by the volume concentration. Finally, the effective density in dispersion with solvent was defined by the following equation [50]. The equation was proposed for agglomerated metal oxide particles. The sample in this work is not agglomerated metal oxide, but swelling polymer. However, the same equation could be applied to estimate the effective density of solvated polymer shell particle [51]. Hence, we have applied the equation to determine the effective density of CL-PAA.

$$\begin{aligned} \rho_{{{\text{Eff}}\_{\text{in}}\,{\text{dispersion}}\,{\text{with}}\,{\text{solv}}{.}}} & = \frac{{\left( {\rho_{poly} V_{poly} } \right) + \left( {\rho_{solv} V_{solv} } \right)}}{{V_{total} }} \\ & = \frac{{\left( {1.43 \times \frac{\pi }{6} 0.92^{3} } \right) + \left( {1.00 \times \frac{\pi }{6} \left( {1.62^{3 } - 0.92^{3} } \right)} \right)}}{{\frac{\pi }{6} 1.62^{3} }} \\ & = {1}.0{8}\,{\text{g}}/{\text{mL}} \\ \end{aligned}$$

Here ρ_Eff in dispersion with solv._: effective density in dispersion with solvent, ρ_poly_: density of polymer (= effective density of polymer), ρ_solv_: density of solvent (= density of water), *V*_poly_: volume of polymer, *V*_solv_: volume of solvent, *V*_total_: volume of swelled polymer (= *V*_poly_ + *V*_solv_).

The MMAD was determined as a mass median value of size distribution measured by Low-Volume Air Sampler (Model AN-200, Andersen Type, TOKYO DYLEC CORP., Tokyo, Japan). The effective density of aerosol condition was also determined by dividing the mass concentration by the volume concentration. The mass concentration in air was determined from the filtered weight of particles and the volume of air. The volume concentration was determined from the MMAD and the number concentration measured by a particle counter (KC-52, RION Co., Ltd., Tokyo, Japan).

The bulk polymer and the dispersed polymer in the solution were seen through the scanning electron microscopy (SEM) by HITACHI S-4500 (Hitachi, Ltd., Tokyo, Japan).

Particle preparations in this experiment were tested for endotoxin using gel clot endotoxin assay kit (Toxin Sensor™) (Gen Script USA Inc., Piscataway, NJ, USA) according to the manufacture’s instruction. The labeled lysate reagent sensitivity in the gel-clot methods of the kit we used was 0.25 EU/mL (a brief summary of the method used is shown in Additional file 3).

### Animals

Male Fischer 344 rats (8 weeks old) were purchased from Charles River Laboratories International, Inc. (Kanagawa, Japan) and acclimated for 2 weeks. They housed under a light, temperature and humidity-controlled room (light/dark 12 h/12 h cycle, 20–25 °C, 40–70% with ventilation 15 times/hour) using Labo Flake (CLEA Japan, Inc., Tokyo, Japan) as bedding, in the Laboratory Animal Research Center of the University of Occupational and Environmental Health, Japan with free access to a commercial diet (CE-2) (CLEA Japan, Inc., Tokyo, Japan) and water. All procedures and animal handling were done according to the guidelines described in the Japanese Guide for the Care and Use of Laboratory Animals as approved by the Animal Care and Use Committee, University of Occupational and Environmental Health, Japan (animal studies ethics clearance proposal number; AE17-009).

### Intratracheal instillation

Doses of 0.2 mg (0.8 mg/kg BW) and 1.0 mg (4.0 mg/kg BW) of CL-PAA suspended in 0.4 ml distilled water were administered to rat lungs (12 weeks old) in single intratracheal instillations. The control group received distilled water.

We performed intratracheal instillation studies of varieties of respirable chemicals such as nanomaterials [16, 17, 52, 53], man-made mineral fibers [54] and asbestos [55], in order to examine the pulmonary toxicity of these respirable chemicals. Although the intratracheal instillation studies, it was considered that the low and high dosages (0.8 mg/kg BW and 4.0 mg/kg BW, respectively) were approximately the minimum and maximum doses needed for evaluating the pulmonary toxicity of respirable chemicals in our experiment. The low dose is around the minimum dose at which respirable chemicals with high pulmonary toxicity. Previously, rats, which were a different species to this experiment, were received at 0.8 mg/kg BW of NiO nanoparticles by intratracheal instillation, and there were observed mild neutrophil inflammation [12]. It was considered that the dose of 4 mg/kg BW was the maximum dose without overload in intratracheal instillation of respirable chemicals. In our previous report, doses in excess of 4 mg/kg BW induced pulmonary excess inflammation and the biological half time of nanoparticles was delayed [56]. It was reported that a clearance of alveolar macrophages was delayed between 1 mg/rat (4 mg/kg BW in our experiment is equivalent to 1 mg/rat) and 3 mg/rat of lung deposition in toner studies [57, 58], and it was shown that 1 to 3 mg/rat is the threshold for overload. Based on these data, it was speculated that if exposure to doses was above 1 mg/rat, toxicity from the excessive dose might occur as well as pulmonary toxicity by the chemicals themselves. The dose of 4.0 mg/kg BW as the lung burden of respirable chemical substances after intratracheal instillation may correspond to a period of approximately 1.8 years of inhalation at a concentration of 3 mg/m^3^ (the maximum concentration for humans of inhalable dust other than crystalline silica (working time 8 h/day, 5 days/week)), respectively, defined by the American Conference of Governmental Industrial Hygienists (ACGIH). If the accumulative exposure in workers who handle CL-PAA is extrapolated from this dosage, that may also equate to a period of approximately 529 days (1.45 years) of inhalation at 2.1 mg/m^3^ in maximum personal exposure concentration of respirable dust (lung weight in rat: 2 g, lung weight in human: 1000 g, tidal volume in human: 0.625 L, respiratory rate: 15/min, exposure time in day: 8 h, deposition fraction: 10%).

### Animals following intratracheal instillation

There were 5 rats in each exposure and control group at each time point. Animals were dissected at 3 days, 1 week, 1 month, 3 months and 6 months after intratracheal instillation under anesthesia with isoflurane (Pfizer Japan, Tokyo, Japan) inhalation. Body and lung weights were measured, then, at autopsy, blood was removed from the abdominal aorta and the lung was perfused with normal saline. The right lungs were repetitively inflated with normal saline under a pressure of 20 cm H_2_O, following fluid recovery two times, while the left main bronchus was clamped. Between 7 and 14 mL of the recovered fluid (BALF) was collected in collection tubes by free fall, and then the right and left lungs were divided. The third lobes of the right lungs after recovery of BALF were stored at -80 °C and used for measurement for HO-1, and RNA extraction and cDNA microarray. The left lungs were inflated and fixed by 10% formaldehyde under a pressure of 25 cm H2O for use in histopathological evaluation.

### Cytospin analysis of inflammatory cells and measurement of LDH in BALF

BALF was centrifuged at 400 g at 4 °C for 15 min, and the supernatant was transferred to a new tube for measurement of LDH and cytokines, and part of that was stored at − 80 °C for measurement for chemokine. The pellets were washed by suspension with polymorphonuclear leukocyte (PMN) Buffer (137.9 mM NaCl, 2.7 mM KCl, 8.2 mM Na_2_HPO_4_, 1.5 mM KH_2_PO_4_ and 5.6 mM C_6_H_12_O_6_) and centrifuged at 400 g at 4 °C for 15 min. After removal of the supernatant, the pellets were resuspended with 1 mL of PMN Buffer. The number of cells in BALF was counted by ADAM-MC (AR BROWN CO., LTD, Tokyo, Japan), and the cells were splashed on a glass slide using cytospin, fixed and stained with Diff-Quik (Sysmex CO., Kobe, Hyogo, Japan). Diff-Quik staining can distinguish various cells including neutrophils or eosinophils, where eosinophils are characterized by the fact that their granules in the cytoplasm are prominent pink, whereas neutrophils’ cytoplasm are clear, pale blue to faintly basophilic with a fine grainy texture. In addition, the nuclei of eosinophils are less condensed and lobulated than those of neutrophils [59]. Then the number of neutrophils and alveolar macrophages were counted by microscopic observation. The released LDH activity in the BALF supernatant was measured by a Cytotoxicity Detection Kit^PLUS^ (LDH) (Roche Diagnostics GmbH, Mannheim, Nordrhein-Westfalen, Germany) according to the manufacturer’s instructions. LDH activity was estimated using a standard curve obtained from known concentrations of recombinant LDH from rabbit muscle (Oriental Yeast Co., ltd., Tokyo, Japan).

### Measurement of chemokines in BALF and HO-1 in lung tissue

Concentrations of CINC-1, CINC-2 and CXCL5 in BALF were measured by ELISA kits, #RCN100, #RCN200 (R&D Systems, Minneapolis, MN, USA), and LS-F23176 (LSBio, Seattle, WA, USA), respectively. All measurements were performed according to the manufacturer’s instructions. The third lobes of the right lungs were homogenized with T-PER tissue protein extraction reagent (Thermo Scientific Inc., Rockford, IL, USA) including protein inhibitor cocktails (P8340, Sigma-Aldrich, St. Louis, MO, USA) and cOmplete Mini (Roche Diagnostics GmbH, Mannheim, Nordrhein-Westfalen, Germany), and then centrifuged (20,400*g* at 4 °C for 10 min). The protein concentration of the supernatant was measured by Pierce 660 nm Protein Assay Reagent (Thermo Scientific Inc., Rockford, IL, USA), using bovine serum albumin as a standard. The total protein concentration was adjusted to a final concentration of 500 mg/mL for measuring the HO-1 by the ELISA kit, ADI-EKS-810A (Enzo Life Sciences, Farmingdale, NY, USA).

### Total RNA extraction

Total RNA extraction was performed as described previously [31], using the third lobes of the right lungs stored at − 80 °C. Briefly, the third lobes of the right lungs of the control group and 1.0 mg of the CL-PAA-exposure group at 1 month after the instillation were homogenized, and total RNA was extracted using a miRNAeasy Mini Kit (Qiagen, Hilden, Nordrhein-Westfalen, Germany). The RNA quantification and quality check were performed using a NanoDrop 2000 spectrophotometer (Thermo Fisher Scientific Inc., Waltham, MA, USA) and a Bioanalyzer 2100 (Agilent Technologies, Santa Clara, CA, USA), respectively.

### Microarray analysis

Microarray analysis was performed as described previously [31]. Briefly, A 3D-Gene Rat Oligo Chips 20K (version 1.1) (Toray Industries, Tokyo, Japan) containing 20,174 genes was used for a 3D-Gene array system (Toray Industries, Tokyo, Japan). For microarray analysis, total RNA extracted from the lungs of the five rats in the control group or the CLL-PAA-high dose group were mixed in equal amounts to make one sample, respectively. One μg of total RNA of each group at 1 month was labeled with Cy5 (GE Healthcare, Buckinghamshire, UK) and amplified by the use of an Amino Allyl MessageAMP II aRNA Amplification Kit (Applied Biosystems, CA, USA). The labeled antisense RNA (aRNA) was hybridized at 37 °C for 16 h according to the supplier’s protocols [60]. The obtained hybridization signals were scanned using a 3D-Gene Scanner (Toray Industries Inc., Tokyo, Japan), and analyzed by 3D-Gene Extraction software (Toray Industries Inc., Tokyo, Japan). The detected signals per microarray were globally normalized, such that the median of the detected signal intensity was adjusted to 25. Gene upregulation or downregulation was defined as having a normalized intensity ratio in which lungs exposed to CL-PAA were 2 times higher or 0.5 times lower than control lungs, respectively. The function of the enhanced 618 expression genes and the KEGG pathway enrichment were analyzed using of Database for Annotation Visualization and Integrated Discovery (DAVID) 6.8 [61].

### Micro-CT imaging

The interval between micro-CT imaging and sacrifice was within a few hours to a few days. For example, at the time of dissection at 3 days after the instillation, micro-CT scan was performed a few hours before it. In the same way, at the time of dissection at 1 week, 1 month, 3 months or 6 months after the instillation, micro-CT scan was performed within 12 h, 24 h, and a few days, respectively. Micro CT imaging was performed on 3 of the 5 animals in each group. The X-ray micro-CT system (CosmoScan GX, Rigaku Co., Tokyo, Japan) was operated with the following parameters: a tube voltage of 90 kV, a tube current of 88 µA, chest CT, 60 $\times$ 40 mm field of view (FOV) (the voxel matrix: $512 \times 512 \times 512$ µm, and the voxel size: $120 \times 120 \times 120$ µm). The lungs were scanned in the prone position under anesthetization with inhalation of mixed sevoflurane (Pfizer Japan, Tokyo, Japan) and oxygen through a nose cone. The exposure time was 4.0 min, and images were retrospectively gated at the inspiration breathing phase with an average whole body exposure of 161.9 mGy/scan.

### Histopathology and immunohistochemistry

Formaldehyde-fixed lung tissue was embedded in paraffin, sectioned at a thickness of 4 μm, and then stained with hematoxylin and eosin (HE) and Masson trichrome (MT) staining. The inflammatory cell infiltration score [31] or the Ashcroft score [62] were performed to evaluate lung inflammation or fibrosis, respectively, according to previous reports. Briefly, as to the inflammatory cell infiltration score, the severity of histological changes in the lungs were scored as none (0), minimal (0.5), mild (1), moderate (2), or severe (3). The score is calculated with a following equation. Σ (grade × number of animals with grade). As to the Ashcroft score, lung sections were assessed by the grade from 0 (normal lung) to 8 (most severe fibrosis), and the grades were summed up and divided by the number of fields. Immunostaining for CXCL5 was performed with rabbit anti-mouse CXCL5 polyclonal antibody (1:200 dilution, bs-2549R; Bioss Inc., Woburn, MA, USA), while using the lung tissue samples from the 1.0 mg CL-PAA-exposure group of one month after intratracheal instillation. The slides were assessed for histological changes by a board-certified pathologist.

### Statistical analysis

Statistical analysis was carried out using JMP® Pro software (JMP Version 14.2.0, SAS Institute Inc., Cary, NC, USA). *P* values < 0.05 were considered statistically significant. To detect individual differences between those exposed to the cross-linked polyacrylate samples and the controls, one-way analysis of variance (ANOVA) followed by Dunnett’s tests were used appropriately. To detect individual difference among the exposed groups, one-way or two-way ANOVA followed by Turkey's test was used to appropriately.

## Supplementary Information

**Additional file 1: Supplemental Table 1. (A)** Description of genes related to ‘inflammatory response’ among 788 genes upregulated ≧ 2-fold. **(B)** Description of genes related to ‘immune response’ among 788 genes upregulated ≧ 2-fold. **(C)** Description of genes related to ‘response to oxidative stress’ among 788 genes upregulated ≧ 2-fold. **Supplemental Table 2. (A)** Description of genes related to ‘epithelial mesenchymal transition’ among 788 genes upregulated ≧ 2-fold. **(B)** Description of genes related to ‘positive regulation of epithelial mesenchymal transition immune response’ among 788 genes upregulated ≧ 2-fold. **Supplemental Table 3.** Signaling pathways of differentially expressed upregulated genes at 1 month after exposure to 1.0 mg-CL-PAA (p < 0.05 and gene counts ≧ 2). 1): “Count” means the number of genes involved in the term. 2): “%” means percentage of “involved genes” / “total genes.” 3) and 4): Fisher’s exact test and modified Fisher’s exact test is adopted to measure the gene-enrichment in annotation terms, respectively. The total number of genes analyzed was 618 genes in this analysis. **Supplemental Table 4.** The listed genes of Supplemental Figure 2 (B).

**Additional file 2: Supplemental Figure 1.** Comparison with CINC-1 **(A)** and CINC-2 **(B)** concentration in BALF between CL-PAA, NiO and CeO_2_ particles during observation period. **Supplemental Figure 2.** KEGG pathways. **(A)** Cytokine-cytokine receptor interaction. **(B)** Chemokine signaling pathway.

**Additional file 3: **A brief summary of the method used and the result of endotoxin measurement of the particle preparations in this experiment.

## Data Availability

The datasets during and/or analyzed during the current study are available from the corresponding author on reasonable request.

## Abbreviations

PHMG-p: Polyhexamethyleneguanidine phosphate
ARDS: Acute respiratory distress syndrome
8 h-TWA: 8 Hour-time weighted average
CL-PAA: Cross-linked polyacrylic acid
PAA: Polyacrylic acid
Mw: Weight average molecular weight
FFF-MALS: Multiangle light scattering coupled with field flow fractionation
DLS: Dynamic light scattering
MMAD: Mass median aerodynamic diameter
SEM: Scanning electron microscopy
LDH: Lactate dehydrogenase
BALF: Bronchoalveolar lavage fluid
CINC: Cytokine-induced neutrophil chemoattractant
CXCL5: C-X-C motif chemokine 5
HO-1: Heme oxygenase-1
CXCL6: C-X-C motif chemokine 6
EMT: Epithelial to mesenchymal transition
KEGG: Kyoto Encyclopedia of Genes and Genomes
NiO: Nickel oxide
CeO_2_: Cerium dioxide
MWCNT: Multi-walled carbon nanotube
ELR: Glutamate-leucine-arginine
CCL22: C–C motif chemokine 22
CCL17: C–C motif chemokine 17
ACGIH: The American Conference of Governmental Industrial Hygienists
TiO2: Titanium dioxide
SR-As: The class A scavenger receptors
PMN: Polymorphonuclear leukocyte
FOV: Field of view
HE: Hematoxylin and eosin
MT: Masson trichrome
