# Supplementary material for: Inflammogenic effect of polyacrylic acid in rat lung following intratracheal instillation
Source: Part Fibre Toxicol. 2022 Jan 21;19:8. doi: 10.1186/s12989-022-00448-z (PMC8780717; doi:10.1186/s12989-022-00448-z)
Supplement: Supplementary file 1 — Additional file 1: Supplemental Table 1. (A) Description of genes related to ‘inflammatory response’ among 788 genes upregulated ≧ 2-fold. (B) Description of genes related to ‘immune response’ among 788 genes upregulated ≧ 2-fold. (C) Description of genes related to ‘response to oxidative stress’ among 788 genes upregulated ≧ 2-fold. Supplemental Table 2. (A) Description of genes related to ‘epithelial mesenchymal transition’ among 788 genes upregulated ≧ 2-fold. (B) Description of genes related to ‘positive regulation of epithelial mesenchymal transition immune response’ among 788 genes upregulated ≧ 2-fold. Supplemental Table 3. Signaling pathways of differentially expressed upregulated genes at 1 month after exposure to 1.0 mg-CL-PAA (p < 0.05 and gene counts ≧ 2). 1): “Count” means the number of genes involved in the term. 2): “%” means percentage of “involved genes” / “total genes.” 3) and 4): Fisher’s exact test and modified Fisher’s exact test is adopted to measure the gene-enrichment in annotation terms, respectively. The total number of genes analyzed was 618 genes in this analysis. Supplemental Table 4. The listed genes of Supplemental Figure 2 (B). [file 12989_2022_448_MOESM1_ESM.docx]

**Supplemental Table 1. (A) Description of genes related to ‘inflammatory response’ among 788 genes upregulated ≧ 2-fold. (B) Description of genes related to ‘immune response’ among 788 genes upregulated ≧ 2-fold. (C) Description of genes related to ‘response to oxidative stress’ among 788 genes upregulated ≧ 2-fold.**

| **(A) Gene symbol** | **Gene description** | **Fold change** |
| --- | --- | --- |
| **Inflammatory response** | | |
| **GO: 0006954** | | |
| ***CXCL6*** | ***Chemokine (C-X-C motif) ligand 6*** | **69.02** |
| ***CXCL11*** | ***Chemokine (C-X-C motif) ligand 11*** | **33.73** |
| ***CCL7*** | ***Chemokine (C-C motif) ligand 7*** | **32.69** |
| ***CCL2*** | ***Chemokine (C-C motif) ligand 2*** | **30.93** |
| ***CXCL13*** | ***Chemokine (C-X-C motif) ligand 13*** | **29.57** |
| ***CCL1*** | ***Chemokine (C-C motif) ligand 1*** | **20.32** |
| ***CCL9*** | ***Chemokine (C-C motif) ligand 9*** | **16.24** |
| ***CXCL10*** | ***Chemokine (C-X-C motif) ligand 10*** | **13.51** |
| ***CCL12*** | ***Chemokine (C-C motif) ligand 12*** | **12.64** |
| ***Chi3l1*** | ***chitinase 3 like 1*** | **9.36** |
| ***C4a*** | ***complement component 4A***  ***(Rodgers blood group)*** | **6.87** |
| ***CXCL2*** | ***Chemokine (C-X-C motif) ligand 2*** | **6.46** |
| ***S100a8*** | ***S100 calcium binding protein A8*** | **5.78** |
| ***Tnfrsf9*** | ***tumor necrosis factor receptor superfamily, member 9*** | **4.91** |
| ***CCL22*** | ***Chemokine (C-C motif) ligand 22*** | **4.46** |
| ***Ncf1*** | ***neutrophil cytosolic factor 1*** | **4.35** |
| ***Ptafr*** | ***platelet-activating factor receptor*** | **3.61** |
| ***Ppbp*** | ***pro-platelet basic protein*** | **3.50** |
| ***Serpina3n*** | ***serine (or cysteine) peptidase inhibitor,***  ***clade A, member 3N*** | **3.33** |
| ***Il24*** | ***interleukin 24*** | **3.17** |
| ***Ccr1*** | ***chemokine (C-C motif) receptor 1*** | **3.15** |
| ***Il6*** | ***interleukin 6*** | **3.09** |
| ***Tnfrsf4*** | ***tumor necrosis factor receptor superfamily, member 4*** | **3.08** |
| ***Tnf*** | ***tumor necrosis factor*** | **3.03** |
| ***Elf3*** | ***E74-like factor 3*** | **3.02** |
| ***CCL17*** | ***Chemokine (C-C motif) ligand 17*** | **2.96** |
| ***Pf4*** | ***platelet factor 4*** | **2.91** |
| ***Cd14*** | ***CD14 molecule*** | **2.85** |
| ***CXCL9*** | ***Chemokine (C-X-C motif) ligand 9*** | **2.83** |
| ***Csf1*** | ***colony stimulating factor 1*** | **2.81** |
| ***Ltb4r*** | ***leukotriene B4 receptor*** | **2.81** |
| ***CCL20*** | ***Chemokine (C-C motif) ligand 20*** | **2.76** |
| ***Ms4a2*** | ***membrane spanning 4-domains A2*** | **2.75** |
| ***CCL3*** | ***Chemokine (C-C motif) ligand 3*** | **2.71** |
| ***Tac4*** | ***tachykinin 4 (hemokinin)*** | **2.65** |
| ***Il18*** | ***interleukin 18*** | **2.61** |
| ***Ccr5*** | ***chemokine (C-C motif) receptor 5*** | **2.60** |
| ***Cybb*** | ***cytochrome b-245, beta polypeptide*** | **2.58** |
| ***Tlr2*** | ***toll-like receptor 2*** | **2.58** |
| ***Aif1*** | ***allograft inflammatory factor 1*** | **2.47** |
| ***Havcr2*** | ***hepatitis A virus cellular receptor 2*** | **2.46** |
| ***Slc11a1*** | ***solute carrier family 11 member 1*** | **2.35** |
| ***Nlrp3*** | ***NLR family, pyrin domain containing 3*** | **2.34** |
| ***Chst1*** | ***carbohydrate (keratan sulfate Gal-6) sulfotransferase 1*** | **2.27** |
| ***Cnr2*** | ***cannabinoid receptor 2*** | **2.25** |
| ***Cx3cl1*** | ***chemokine (C-X3-C motif) ligand 1*** | **2.23** |
| ***CCL4*** | ***Chemokine (C-C motif) ligand 4*** | **2.14** |
| ***Hck*** | ***hemopoietic cell kinase*** | **2.10** |
| ***CCL19*** | ***Chemokine (C-C motif) ligand 19*** | **2.10** |
| ***Tlr6*** | ***toll-like receptor 6*** | **2.04** |
| ***Cxcr3*** | ***chemokine (C-X-C motif) receptor 3*** | **2.03** |
| ***Olr1*** | ***oxidized low density lipoprotein (lectin-like) receptor 1*** | **2.01** |

| **(B) Gene symbol** | **Gene description** | **Fold change** |
| --- | --- | --- |
| **Immune response** | | |
| **GO: 0006955** | | |
| ***CXCL5*** | ***Chemokine (C-X-C motif) ligand 5*** | **69.02** |
| ***CXCL11*** | ***Chemokine (C-X-C motif) ligand 11*** | **33.73** |
| ***CXCL13*** | ***Chemokine (C-X-C motif) ligand 13*** | **29.57** |
| ***CXCL10*** | ***Chemokine (C-X-C motif) ligand 10*** | **13.51** |
| ***C4bpa*** | ***complement component 4 binding protein, alpha*** | **9.30** |
| ***C6*** | ***complement component 6*** | **7.98** |
| ***Mcpt1l1*** | ***mast cell protease 1-like 1*** | **7.88** |
| ***Mcpt2*** | ***mast cell protease 2*** | **7.88** |
| ***Sectm1a*** | ***secreted and transmembrane 1A*** | **7.15** |
| ***Fcar*** | ***Fc fragment of IgA receptor*** | **6.99** |
| ***CXCL2*** | ***Chemokine (C-X-C motif) ligand 2*** | **6.46** |
| ***Fcgr2b*** | ***Fc fragment of IgG, low affinity IIb, receptor*** | **5.82** |
| ***C1qb*** | ***complement component 1, q subcomponent, B chain*** | **5.69** |
| ***Tnfrsf9*** | ***tumor necrosis factor receptor superfamily, member 9*** | **4.91** |
| ***Osm*** | ***oncostatin M*** | **4.32** |
| ***Clec4e*** | ***C-type lectin domain family 4, member E*** | **3.55** |
| ***Ppbp*** | ***pro-platelet basic protein*** | **3.50** |
| ***RGD1565617*** | ***similar to Ig variable region, light chain*** | **3.33** |
| ***CXCL1*** | ***Chemokine (C-X-C motif) ligand1*** | **3.33** |
| ***Ctss*** | ***cathepsin S*** | **3.29** |
| ***Il24*** | ***interleukin 24*** | **3.17** |
| ***Enpp3*** | ***ectonucleotide pyrophosphatase/phosphodiesterase 3*** | **3.15** |
| ***Ccr1*** | ***chemokine (C-C motif) receptor 1*** | **3.15** |
| ***Blnk*** | ***B-cell linker*** | **3.12** |
| ***Tnfrsf4*** | ***tumor necrosis factor receptor superfamily, member 4*** | **3.08** |
| ***Tnf*** | ***tumor necrosis factor*** | **3.03** |
| ***Pf4*** | ***platelet factor 4*** | **2.91** |
| ***CXCL9*** | ***Chemokine (C-X-C motif) ligand 9*** | **2.83** |
| ***RT1-DMa*** | ***RT1 class II, locus DMa*** | **2.75** |
| ***Ms4a2*** | ***membrane spanning 4-domains A2*** | **2.75** |
| ***Ltb*** | ***lymphotoxin beta*** | **2.64** |
| ***Ccr5*** | ***chemokine (C-C motif) receptor 5*** | **2.60** |
| ***Tlr2*** | ***toll-like receptor 2*** | **2.58** |
| ***RT1-DMb*** | ***RT1 class II, locus DMb*** | **2.40** |
| ***RT1-Bb*** | ***RT1 class II, locus Bb*** | **2.38** |
| ***Mcpt10*** | ***mast cell protease 10*** | **2.32** |
| ***Tnfsf13*** | ***tumor necrosis factor superfamily member 13*** | **2.29** |
| ***Ctsh*** | ***cathepsin H*** | **2.26** |
| ***Ccr10*** | ***chemokine (C-C motif) receptor 10*** | **2.24** |
| ***Cx3cl1*** | ***chemokine (C-X3-C motif) ligand 1*** | **2.23** |
| ***Lst1*** | ***leukocyte specific transcript 1*** | **2.15** |
| ***CCL4*** | ***Chemokine (C-C motif) ligand4*** | **2.15** |
| ***Cd274*** | ***CD274 molecule*** | **2.12** |
| ***LOC691603*** | ***similar to mast cell protease 9*** | **2.12** |
| ***Irf8*** | ***interferon regulatory factor 8*** | **2.08** |
| ***Lcp2*** | ***lymphocyte cytosolic protein 2*** | **2.05** |
| ***Tlr6*** | ***toll-like receptor 6*** | **2.04** |

| **(C) Gene symbol** | **Gene description** | **Fold change** |
| --- | --- | --- |
| **Response to oxidative stress** | | |
| **GO: 0006979** | | |
| ***Mt3*** | ***metallothionein 3*** | **53.16** |
| ***Lpo*** | ***lactoperoxidase*** | **33.15** |
| ***Lcn2*** | ***lipocalin 2*** | **30.71** |
| ***Duox1*** | ***dual oxidase 1*** | **5.01** |
| ***Hmox1*** | ***heme oxygenase 1*** | **4.62** |
| ***Apoe*** | ***apolipoprotein E*** | **4.39** |
| ***Prdx5*** | ***peroxiredoxin 5*** | **3.25** |
| ***Sod2*** | ***superoxide dismutase 2, mitochondrial*** | **3.05** |
| ***Mmp9*** | ***matrix metallopeptidase 9*** | **2.60** |

**Supplemental Table 2. (A) Description of genes related to ‘epithelial mesenchymal transition’ among 788 genes upregulated ≧ 2-fold. (B) Description of genes related to ‘positive regulation of epithelial mesenchymal transition immune response’ among 788 genes upregulated ≧ 2-fold.**

| **(A) Gene symbol** | **Gene description** | **Fold change** |
| --- | --- | --- |
| **Epithelial mesenchymal transition** | | |
| **GO: 0001837** | | |
| ***Tgfbr1*** | ***transforming growth factor, beta receptor 1*** | **3.14** |

| **(B) Gene symbol** | **Gene description** | **Fold change** |
| --- | --- | --- |
| **Positive regulation of epithelial mesenchymal transition** | | |
| **GO: 0010718** | | |
| ***Ezh2*** | ***enhancer of zeste 2 polycomb repressive complex 2 subunit*** | **2.37** |
| ***Dab2*** | ***disabled 2, mitogen-responsive phosphoprotein*** | **2.31** |
| ***Col1a1*** | ***collagen, type I, alpha 1*** | **2.16** |

**Supplemental Table 3. Signaling pathways of differentially expressed upregulated genes at 1 month after exposure to 1.0 mg-CL-PAA (*p* <0.05 and gene counts ≧ 2).**

| **Category** | **Term** | **Count** | **%** | **P-Value** | **Benjamini** |
| --- | --- | --- | --- | --- | --- |
| **KEGG_PATHWAY** | **Cytokine-cytokine receptor interaction** | **31** | **5** | **7.00E-12** | **1.30E-09** |
| **KEGG_PATHWAY** | **Chemokine signaling pathway** | **29** | **4.7** | **1.10E-11** | **1.30E-09** |
| **KEGG_PATHWAY** | **Rheumatoid arthritis** | **21** | **3.4** | **2.00E-11** | **1.50E-09** |
| **KEGG_PATHWAY** | **Staphylococcus aureus infection** | **16** | **2.6** | **2.40E-10** | **1.40E-08** |
| **KEGG_PATHWAY** | **Lysosome** | **22** | **3.6** | **2.10E-09** | **9.80E-08** |
| **KEGG_PATHWAY** | **Pertussis** | **16** | **2.6** | **2.20E-08** | **8.60E-07** |
| **KEGG_PATHWAY** | **Complement and coagulation cascades** | **15** | **2.4** | **1.40E-07** | **4.60E-06** |
| **KEGG_PATHWAY** | **Tuberculosis** | **22** | **3.6** | **1.20E-06** | **3.60E-05** |
| **KEGG_PATHWAY** | **Phagosome** | **22** | **3.6** | **3.20E-06** | **8.40E-05** |
| **KEGG_PATHWAY** | **Chagas disease (American trypanosomiasis)** | **14** | **2.3** | **8.10E-05** | **1.90E-03** |
| **KEGG_PATHWAY** | **Toll-like receptor signaling pathway** | **13** | **2.1** | **1.30E-04** | **2.70E-03** |
| **KEGG_PATHWAY** | **Protein digestion and absorption** | **12** | **1.9** | **2.40E-04** | **4.80E-03** |
| **KEGG_PATHWAY** | **Mineral absorption** | **8** | **1.3** | **3.40E-04** | **6.20E-03** |
| **KEGG_PATHWAY** | **Legionellosis** | **9** | **1.5** | **7.30E-04** | **1.20E-02** |
| **KEGG_PATHWAY** | **NOD-like receptor signaling pathway** | **9** | **1.5** | **7.30E-04** | **1.20E-02** |
| **KEGG_PATHWAY** | **Amino sugar and nucleotide sugar metabolism** | **8** | **1.3** | **1.20E-03** | **1.80E-02** |
| **KEGG_PATHWAY** | **Amoebiasis** | **12** | **1.9** | **1.70E-03** | **2.40E-02** |
| **KEGG_PATHWAY** | **Leishmaniasis** | **9** | **1.5** | **3.40E-03** | **4.50E-02** |
| **KEGG_PATHWAY** | **Malaria** | **8** | **1.3** | **4.20E-03** | **5.20E-02** |
| **KEGG_PATHWAY** | **TNF signaling pathway** | **11** | **1.8** | **4.60E-03** | **5.40E-02** |
| **KEGG_PATHWAY** | **Antigen processing and presentation** | **10** | **1.6** | **6.50E-03** | **7.00E-02** |
| **KEGG_PATHWAY** | **Central carbon metabolism in cancer** | **8** | **1.3** | **6.60E-03** | **7.00E-02** |
| **KEGG_PATHWAY** | **Hematopoietic cell lineage** | **9** | **1.5** | **7.50E-03** | **7.70E-02** |
| **KEGG_PATHWAY** | **Cell cycle** | **11** | **1.8** | **1.30E-02** | **1.30E-01** |
| **KEGG_PATHWAY** | **Osteoclast differentiation** | **11** | **1.8** | **1.40E-02** | **1.40E-01** |
| **KEGG_PATHWAY** | **Systemic lupus erythematosus** | **11** | **1.8** | **1.70E-02** | **1.50E-01** |
| **KEGG_PATHWAY** | **Cell adhesion molecules (CAMs)** | **13** | **2.1** | **1.70E-02** | **1.50E-01** |
| **KEGG_PATHWAY** | **Asthma** | **5** | **0.8** | **2.20E-02** | **1.80E-01** |
| **KEGG_PATHWAY** | **Intestinal immune network for IgA production** | **6** | **1** | **2.30E-02** | **1.90E-01** |
| **KEGG_PATHWAY** | **Transcriptional misregulation in cancer** | **12** | **1.9** | **2.40E-02** | **1.90E-01** |
| **KEGG_PATHWAY** | **Inflammatory bowel disease (IBD)** | **7** | **1.1** | **2.50E-02** | **1.90E-01** |
| **KEGG_PATHWAY** | **Glycosaminoglycan degradation** | **4** | **0.6** | **3.50E-02** | **2.60E-01** |
| **KEGG_PATHWAY** | **Influenza A** | **12** | **1.9** | **3.60E-02** | **2.60E-01** |
| **KEGG_PATHWAY** | **Cytosolic DNA-sensing pathway** | **6** | **1** | **4.50E-02** | **3.10E-01** |
| **KEGG_PATHWAY** | **Type I diabetes mellitus** | **7** | **1.1** | **4.90E-02** | **3.30E-01** |

1): “Count” means the number of genes involved in the term. 2): “%” means percentage of “involved genes” / “total genes.” 3) and 4): Fisher’s exact test and modified Fisher’s exact test is adopted to measure the gene-enrichment in annotation terms, respectively.

The total number of genes analyzed was 618 genes in this analysis.

**Supplemental Table 4. The listed genes of Supplemental Figure 2 (B).**

| **Gene symbol** | **Gene description** |
| --- | --- |
| ***CCL17*** | ***C-C motif chemokine ligand 17*** |
| ***CCL19*** | ***C-C motif chemokine ligand 19*** |
| ***CCL2*** | ***C-C motif chemokine ligand 2*** |
| ***CCL3*** | ***C-C motif chemokine ligand 3*** |
| ***CCL4*** | ***C-C motif chemokine ligand 4*** |
| ***CCL7*** | ***C-C motif chemokine ligand 7*** |
| ***Ccr1*** | ***C-C motif chemokine receptor 1*** |
| ***Ccr10*** | ***C-C motif chemokine receptor 10*** |
| ***CXCL1*** | ***C-X-C motif chemokine ligand 1*** |
| ***CXCL10*** | ***C-X-C motif chemokine ligand 10*** |
| ***CXCL11*** | ***C-X-C motif chemokine ligand 11*** |
| ***CXCL13*** | ***C-X-C motif chemokine ligand 13*** |
| ***CXCL2*** | ***C-X-C motif chemokine ligand 2*** |
| ***CXCL6*** | ***C-X-C motif chemokine ligand 6*** |
| ***Cxcr2*** | ***C-X-C motif chemokine receptor 2*** |
| ***Cxcr3*** | ***C-X-C motif chemokine receptor 3*** |
| ***Cx3cl1*** | ***C-X3-C motif chemokine ligand 1*** |
| ***Gngt2*** | ***G protein subunit gamma transducin 2*** |
| ***Hck*** | ***HCK proto-oncogene, Src family tyrosine kinase*** |
| ***Tiam1*** | ***T-cell lymphoma invasion and metastasis 1*** |
| ***Xcl1*** | ***X-C motif chemokine ligand 1*** |
| ***CCL12*** | ***chemokine (C-C motif) ligand 12*** |
| ***CCL9*** | ***chemokine (C-C motif) ligand 9*** |
| ***Ccr5*** | ***chemokine (C-C motif) receptor 5*** |
| ***Ncf1*** | ***neutrophil cytosolic factor 1*** |
| ***Plcb2*** | ***phospholipase C, beta 2*** |
| ***Pf4*** | ***platelet factor*** |
| ***Ppbp*** | ***pro-platelet basic protein*** |
