# Supplementary material for: Inflammogenic effect of polyacrylic acid in rat lung following intratracheal instillation
Source: Part Fibre Toxicol. 2022 Jan 21;19:8. doi: 10.1186/s12989-022-00448-z (PMC8780717; doi:10.1186/s12989-022-00448-z)

**Supplemental Figure 1. Comparison with CINC-1 (A) and CINC-2 (B) concentration in BALF between CL-PAA, NiO and CeO_2_ particles during observation period.**

| **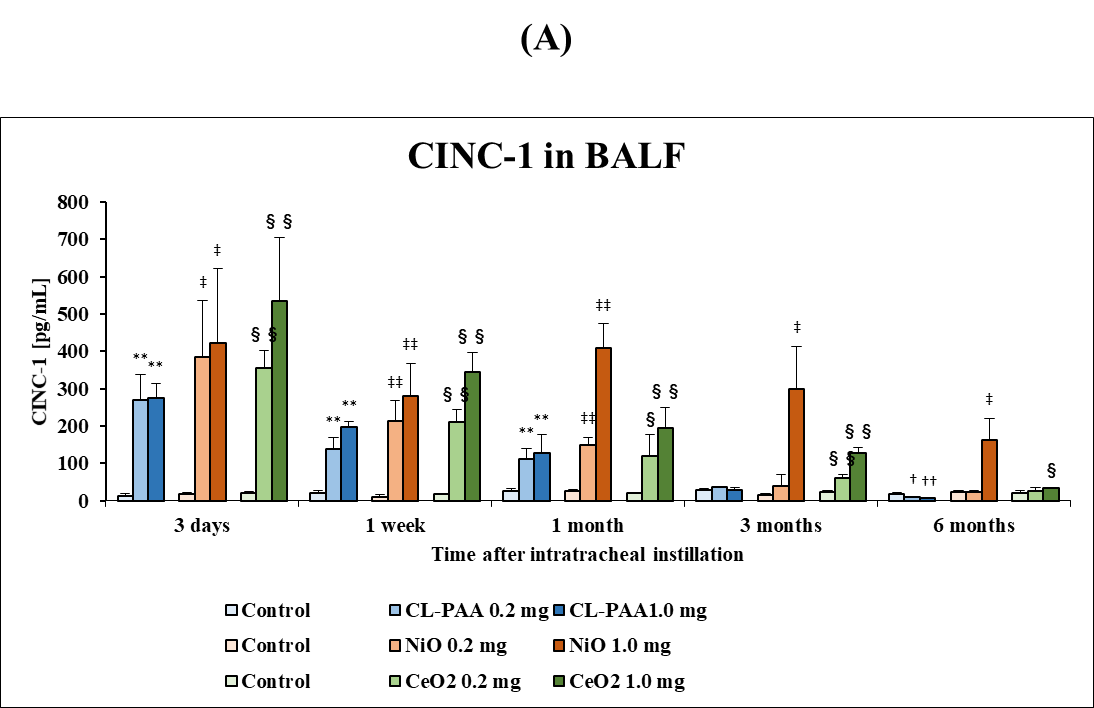** | **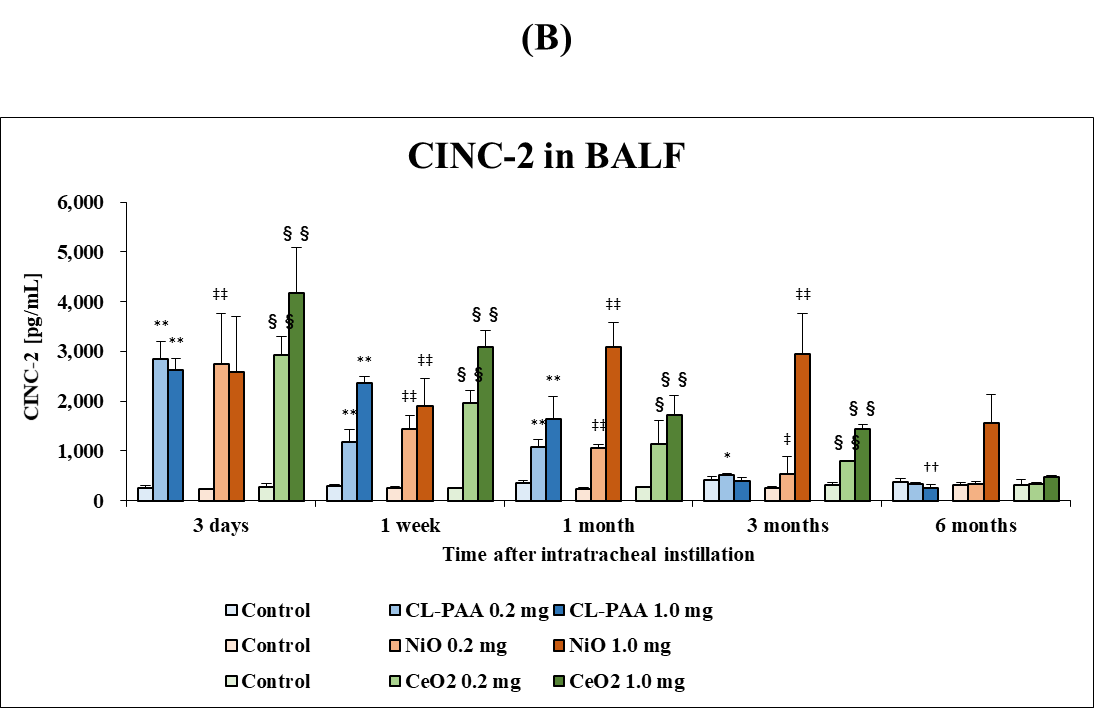** |
| --- | --- |

**Supplemental Figure 2. KEGG pathways.**

**(A) Cytokine-cytokine receptor interaction.**


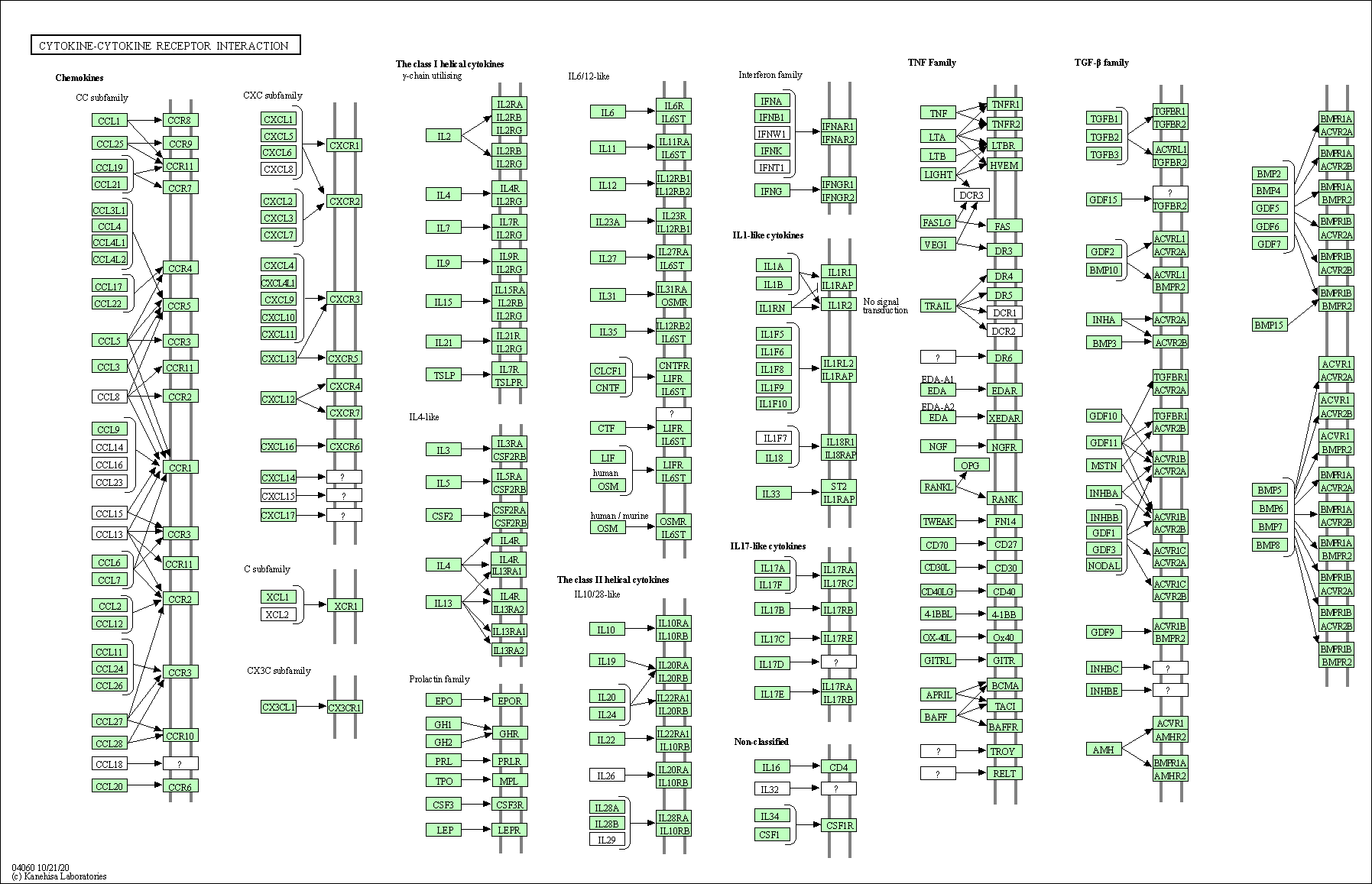


**(B) Chemokine signaling pathway.**


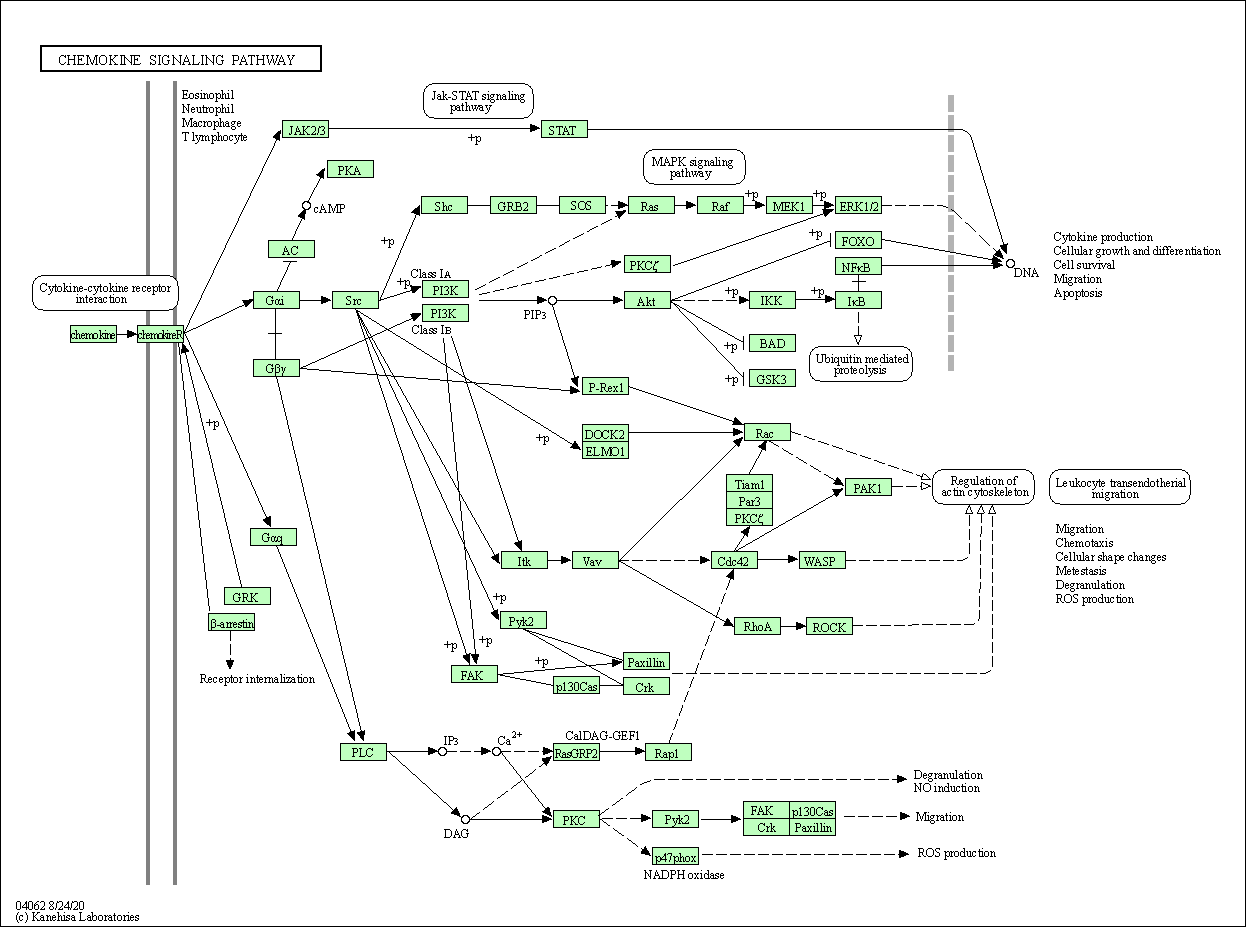

Supplement: Supplementary file 2 — Additional file 2: Supplemental Figure 1. Comparison with CINC-1 (A) and CINC-2 (B) concentration in BALF between CL-PAA, NiO and CeO2 particles during observation period. Supplemental Figure 2. KEGG pathways. (A) Cytokine-cytokine receptor interaction. (B) Chemokine signaling pathway. [file 12989_2022_448_MOESM2_ESM.docx]
