# Supplementary material for: Inflammogenic effect of polyacrylic acid in rat lung following intratracheal instillation
Source: Part Fibre Toxicol. 2022 Jan 21;19:8. doi: 10.1186/s12989-022-00448-z (PMC8780717; doi:10.1186/s12989-022-00448-z)
Supplement: Supplementary file 3 — Additional file 3: A brief summary of the method used and the result of endotoxin measurement of the particle preparations in this experiment. [file 12989_2022_448_MOESM3_ESM.docx]

**Supplemental Material 1.**

A brief summary of the method used and the result of endotoxin measurement of the particle preparations in this experiment are shown below.

We measured endotoxin of the particle preparations in this experiment using gel clot endotoxin assay kit (Toxin Sensor TM) (Gen Script USA Inc., Piscataway, NJ, USA) according to the manufacture’s instruction. This kit is designed to be the simplest semi-quantitative test for gram-negative bacterial endotoxin that conforms to the Food and Drug Administration (FDA) Guideline. This measurement method utilizes that *Limulus* amoebocyte lysate (LAL) gel clot formation based on a multi-step enzymatic reaction initiated by the reaction of endotoxin with the LAL assay solution in a 1:1 ratio. In brief, we simply mix the LAL assay standard solution with the analyte solution of interest in a 1:1 ratio at 37°C and if after 1 hour the combined solution forms a gel, then this is considered positive [1]. In this assay kit our used, the endotoxin level in the positive control sample is equal or higher than 0.25 EU/ml, while in the negative sample is lower than 0.25 EU/ml.

As a result of this test, the combined solution of ours did not form a gel clot and was considered negative.

Reference

1. Scudder J, Ye JY. Limulus amoebocyte lysate test via an open-microcavity optical biosensor.

J Biomed Opt. 2018 Feb;23(2):1-6. doi: 10.1117/1.JBO.23.2.027001.
